# *Campylobacter jejuni* ST353 and ST464 cause localized gut inflammation, crypt damage, and extraintestinal spread during large- and small-scale infection in broiler chickens

**DOI:** 10.1128/aem.01614-24

**Published:** 2025-02-18

**Authors:** Heather M. Chick, Lisa K. Williams, Nick Sparks, Farina Khattak, Paul Vermeij, Inge Frantzen, Mandy Peeters, Jetta J. E. Bijlsma, Daniel John, Timothy Ogunrin, Keioni Essex, Caroline Cayrou, Venkateswarlu Kanamarlapudi, Christopher D. Bayliss, Julian M. Ketley, Thomas J. Humphrey, Steven P. Rushton, Thomas S. Wilkinson

**Affiliations:** 1 Microbiology and Infectious Disease, Institute of Life Science, Swansea University Medical School 151375 , Swansea, United Kingdom; 2 Department of Animal and Agriculture, Hartpury University 12013 , Gloucester, United Kingdom; 3 Scotland’s Rural College (SRUC) Barony Campus 11988 , Dumfries, United Kingdom; 4 Monogastric Science Research Center, Scotland’s Rural College (SRUC) 3123 , Edinburgh, United Kingdom; 5 MSD Animal Health 35956 , Boxmeer, the Netherlands; 6 Genetics, Genomics, and Cancer Sciences, University of Leicester 4488 , Leicester, United Kingdom; 7 Cellular Biology, Institute of Life Science, Swansea University Medical School 151375 , Swansea, United Kingdom; 8 School of Natural and Environmental Sciences, Newcastle University 98458 , Newcastle upon Tyne, United Kingdom; INRS Armand-Frappier Sante Biotechnologie Research Center, Laval, Quebec, Canada

**Keywords:** *in vivo *chicken infection, broiler chicken, *Campylobacter jejuni*, cecal tonsil-derived cytokine production, gut damage, crypt depth, biomarkers, gut health

## Abstract

*Campylobacter* infections in humans and chickens are a significant burden to health services and the poultry industry. In the UK, over 75% of chicken products are *Campylobacter*-positive at retail, but the knowledge of the mechanisms responsible for extraintestinal spread into edible tissues remains incomplete. This work aimed to establish if two chicken-associated lineages of *Campylobacter jejuni*, ST353 and ST464, have the potential for extraintestinal spread. Large- and small-scale chicken colonization trials investigated the infection biology of *C. jejuni* ST353 (three strains) and ST464 (four strains). Both lineages strongly colonized the ileum and ceca and were detected in liver and spleen. *C. jejuni* ST353 and ST464 spleen load were significantly increased compared to *C. jejuni* M1 controls. Immune responses in cecal tonsils exhibited early induction of IFN-γ and suppressed TGFβ at 7 days post-infection with *C. jejuni* ST464. Histochemistry of gut tissue demonstrated significant decreases in intestinal crypt depth in ileal tissue with increasing severity relative to *Campylobacter* lineage, M1 <ST353<ST464. Pairwise correlation analysis confirmed strong interdependencies between “cecal *Campylobacter* load,” “CXCLi1,” “CXCLi2,” and “splenic *Campylobacter* load.” Furthermore, linear discriminant analysis confirmed that cecal tonsil-derived IFNγ, TGFβ, and CXCLi1 could predict splenic invasion at 71% accuracy. This work demonstrates that two chicken specialist *C. jejuni* lineages, ST353 and ST464, cause extraintestinal spread to liver and spleen and modeling suggests distinct routes from ileum and cecum, respectively. Recognition of these two routes of *Campylobacter* extraintestinal spread (ileal/liver and cecal/spleen) provides a better understanding of this food-derived pathogen for academia and the industry.

**IMPORTANCE:**

The UK is self-sufficient in chicken meat production, which remains a cheap and healthy source of dietary protein. However, *Campylobacter* species are present in 75% of raw chicken products at retail sales, resulting in increased human gut infections. Currently, it is not clear which members of the *Campylobacter jejuni* species can leave the digestive tract and reach edible tissues. Using industry-relevant conditions, two *C. jejuni* lineages (ST353 and ST464) isolated from chicken gut and liver were shown to cause infections outside the gut. The underlying mechanisms involve inducing inflammation and gut damage to structures required for cell renewal (crypts) of the intestine. Modeling this data leads to our proposal that *C. jejuni* uses two invasion pathways; one where spread is from ileum to liver and the other between ceca and spleen. Knowledge of these two routes of extraintestinal spread will help the industry develop control measures to improve food biosecurity in poultry.

## INTRODUCTION

*Campylobacter jejuni* represents one of the most significant foodborne pathogens in the United Kingdom, causing approximately 250,000 cases of gastroenteritis annually (1, 2). The annual burden of *Campylobacter* infection has been estimated to be $1.3–$6.8 billion in the US (3) and £0.71 billion in the UK (4) with raw and undercooked poultry muscle and liver shown to be significant sources (5). A retrospective cohort study over 15 years (6) comparing resource costs pre- and post-*Campylobacter* infection found that patients with prescriptions for proton pump inhibitors (12 months before infection) and cephalosporins (within 7 days of infection) had elevated risk of irritable bowel syndrome. These sequelae have led to ~£1.3 million in additional annual National Health Service (NHS) expenditure. *C. jejuni* is responsible for most human cases of campylobacteriosis and is frequently associated with poultry (1, 7). In a recent study, 73.3% of raw chicken products from UK supermarkets were found to be contaminated with *Campylobacter* (8), representing a significant public health risk.

Two major points exist within the chicken production chain where *Campylobacter* containment within the gut is lost resulting in contamination of edible tissues and persistence through the production process (9–11). The first point is “on-farm” where chickens are reared at high stocking densities, in breeds with compromised immunity and with high-stress levels. Multiple studies have shown that broiler chicken breeds have compromised gut barrier integrity (12, 13) leading to extraintestinal spread. Indeed, some studies report that *Campylobacter* strains reach the liver in 70% of fast-growing broilers (14). The second point is “in-abattoir,” where mechanical disruption and evisceration lead to a significant transfer of contaminated gut tissues to edible retail products. The current work focuses on the infection biology of *Campylobacter* extraintestinal spread.

While *Campylobacter* was previously considered a commensal in chickens (15–17), multiple lines of evidence indicate that benign colonization leads to pathogenic infections with observable symptoms and measurable responses in chickens. First, studies have reported that *Campylobacter* causes diarrhea in chickens, resulting in wet and infected litter, which in turn leads to hock burn lesions and pododermatitis (12, 18–21). Second, *Campylobacter* has been found in extraintestinal tissues of poultry, including the liver, muscle, and spleen (9–11, 22), and also expresses numerous virulence factors to aid this process (23). Third, gut microbiome diversity decreases following *Campylobacter* infection (24–26). Finally, these infections lead to dysregulation in gut immunity marked by quantifiable increases in inflammation and a decrease in immune barrier integrity (12, 13, 27, 28). When these factors combine, they lead to *Campylobacter* extraintestinal spread, increasing infection of edible tissues (such as liver and muscle), decreasing food biosecurity, increasing public health threats, and heightened animal welfare concerns.

The chain of events leading to extraintestinal spread in chickens has only been partially characterized. Broiler birds are typically colonized approximately 2–4 weeks after hatching with the principal site of colonization (~10^7^–10^9^ cfu/g) being the ceca (29, 30). However, our previous modeling of *Campylobacter* movement in the gut suggests that the ileum may be the site of initial colonization during early infection (31). Consistent with this, some studies confirm that the ileum is often associated with the greatest damage (13) and spread to the liver, which has a higher incidence with strains found in the upper gut (32). Much of what is known about the extraintestinal spread of *Campylobacter* in chicken has been achieved during *in vivo* experimental infection with a limited set of strains. These include 13126 (14, 32), M1 (12, 32), NCTC11168 (33), NCTC12744 (13, 34), 81–176, and A74C (35). Collectively, these strains are problematic as (i) they do not reflect the full *Campylobacter* species diversity; (ii) they are generalist strains (such as ST21 and ST45), not chicken specialist strains (e.g., ST353 and ST464); and (iii) they are isolates from human disease cases. There is limited work on strains isolated from chickens (36–38) and, in particular, extraintestinal tissues (39).

*Campylobacter* induces a significant immune response in farmed chickens with low doses (≤10^6^ cfu) of *Campylobacter* leading to a coordinated immune response and clearance, whereas higher doses (>10^6^ cfu) (40) are associated with an inability to induce host defense peptides. During the immune response, the Th1 cytokines, IFN-γ, IL-1β, TNFα, IL-6, and IL-2, are induced before Th2 cytokines TGFβ2, IL-10, and IL-4, although differences in the kinetics significantly affect the final response to infection (41, 42). Indeed infection of avian epithelial cells with 100 *Campylobacter* strains demonstrated that each strain had a unique inflammatory profile *in vitro* (43). The level of inflammation is, however, dependent on both chicken breed (12) and *Campylobacter* strain (14). Furthermore, higher levels of inflammation are associated with diarrhea, hock burn lesions, and pododermatitis (14, 24).

Predictive *in silico* models are required to determine how extraintestinal spread is linked to gut inflammation, gut damage, and *Campylobacter* strain. Predictive models of chicken gut health during *Campylobacter* infection have the potential to inform biomarker identification and improve our understanding of infection dynamics. Structural equation modeling (SEM) has been utilized to show that the balance between inflammatory IL-17 and regulatory IL-10 could determine the outcome and time course of *C. jejuni* M1 infections (44). This model also showed that *Campylobacter* load was negatively related to IgY levels in the ileum, suggesting that *Campylobacter* colonization was linked to immune competence (31). In order to broaden and enhance the applicability of these *in silico* models, we performed large- and small-scale *in vivo* chicken trials using seven *Campylobacter* isolates (37, 38) sourced from naturally infected chickens and representing two of the chicken-specialist lineages (ST353 and ST464). Subsequently, bacterial, immunological, and histology markers were determined, and pairwise correlation and linear discriminant analysis (LDA) were used to identify associations between parameters and extraintestinal spread.

## MATERIALS AND METHODS

### *C. jejuni* strains used in this study

This study used a *C. jejuni* strain set (Table S1) consisting of known sequence types with evidence of *in vivo* chicken gut colonization and extraintestinal spread. *C. jejuni* M1 (12, 14, 45) and NCTC11168 (33, 46, 47) were used as positive controls. Three sequence type ST353 and four ST464 strains were used from naturally infected chickens. These strains have been characterized genetically and for *in vitro* invasiveness (37, 38)

### Preparation of *Campylobacter* inoculum

*C. jejuni* strains (Table S1) were stored at –80°C in bead cryopreservation vials (Technical Service Consultants, UK). Strains were resuscitated on Columbia blood agar plates (Oxoid, UK). These cultures were used to prepare lawn plates on blood agar and incubated for 40–48 hours at 41.5°C microaerobically (10% CO_2_, 10% O_2_, 80% N_2_; CampyGen, Oxoid, UK). The lawn plates were harvested by adding 5 mL Mueller–Hinton (MH) broth (Sigma-Aldrich, UK), gently detaching the culture with a sterile spreader and decanting to a container. The suspension was then adjusted with Mueller Hinton broth to an OD_600nm_ of 0.19–0.21 (approximately 1.5 × 10^5^ cfu/mL).

### Chicken infection trials

#### Experiment 1: Large-scale chicken trial (SRUC, UK)

Nine hundred ninety Ross 308 male broiler chicks (*Gallus gallus domesticus*) were housed in a commercial style environmentally controlled building from a day old in a single litter-floor pen. Chicks were reared until 35 days of age. To mimic commercial practice in the UK, birds were fed a wheat soybean-based commercial broiler diet over two phases: the starter phase from days 0 to 21; and the grower phase from days 21 to 35 with the diet manufactured at Target Feeds Ltd (Whitchurch, Shropshire). Food and water were provided *ad libitum* from a circular food hopper and bell drinker. There were 11 treatments (Table S2). Each treatment consisted of a group of 45 chickens (equivalent to a terminal stocking density of 33 kg/m^2^) that were segregated into one pen. Each of the treatments was duplicated, resulting in a total of 22 pens, and arranged in a randomized block design (Fig. S1; Table S3). On day 18, all pens were tested for the presence and absence of *Campylobacter* via boot swabs (1 boot swab/pen = 22 boot swabs in total). On day 21, all birds in treatment groups 1–10 were challenged with ~10^5^ cfu *C*. *jejuni* by direct intubation into the crop via oral gavage with 1 mL of the relevant inoculum. Birds in the uninfected control group (Treatment 11) were intubated with distilled water. On days 28 and 35 (7- and 14-days post-infection [dpi], respectively), 10 birds/pen (out of the 45) were randomly selected and humanely killed (cervical dislocation) with blood and tissue samples taken as described below. The study was terminated on day 35.

#### Experiment 2: Small-scale chicken trial (Boxmeer, Netherlands)

A sub-group of *Campylobacter* strains (Table S2) was used in a small-scale infection experiment at a different laboratory site. These strains were chosen based on their growth characteristics and colonization levels as quantified in Experiment 1. *Campylobacter* M1 was used as a positive control and for correlation with previous findings (12, 21). One hundred twenty male Ross 308 chickens were housed and reared as for the large-scale experiment. The infection protocol was the same as Experiment 1.

### *Campylobacter* tissue load determination

Processing of samples for experiments 1 and 2 was identical where possible, unless stated otherwise. Ten birds from each group (Table S2) were euthanized at 7 and 14 dpi to determine the course of the *Campylobacter* infection. Cecal contents, ileal contents, and sections of the spleen and liver were removed for enumeration of *Campylobacter* spp. Cecal and ileal contents were homogenized in 0.85% NaCl, serially diluted in the same media, and plated on modified charcoal cefoperazone deoxycholate agar plates (mCCDA; Oxoid, UK) for Experiment 1 or on Brilliance CampyCount agar (Oxoid, UK) selective plates for Experiment 2. These plates were incubated at 42°C for 48 hours under microaerobic conditions consisting of 10% CO_2_, 2% H_2_, 5% O_2_, and 80% N_2_ and then enumerated. The atmosphere was generated by both pre-mixed gas and microaerobic atmosphere generation packs due to the space needed. There were no differences between atmosphere generation methods. Five grams of liver or spleen were dipped in ethanol and flamed before being transferred to a stomacher bag containing 45 mL of saline solution, homogenized for 1 minute, and either directly plated or inoculated into modified Exeter broth (48) and incubated microaerobically at 37°C for 48 hours before plating. For enrichment, 10 µL of the enriched broth was plated onto mCCDA and incubated microaerobically at 42°C for 48 hours. Direct liver and spleen samples were plated onto mCCDA and enumerated. Colonies were confirmed by morphology and lack of growth on blood plates grown aerobically.

### Routine bird health monitoring growth and pathology

The bulk weight of birds/pen was measured at 0, 21, 28, and 35 days of age. Weight gains, feed intake, feed conversion ratio, and mortality were calculated for four time periods: days 0–21, 21–28, 28–35, and 0–35. Daily health, culls, and mortality (with cause of death) were recorded, and any unexpected deaths or birds in ill health were subject to postmortem. At the final time point (14 dpi), the presence or absence of hock burn and footpad dermatitis, scores were assessed for every bird using a 0 (no lesions) to 2 (severe lesions) scoring system as described previously (49). However, in this study, the scores are reported (Table S4) as absence (score 0) and presence (sum of score 1 + 2).

### Tissue pathology

In each treatment group, birds were chosen at random, and ileal and cecal tissues were fixed in 4% (wt/vol) paraformaldehyde in phosphate-buffered saline (PBS) and embedded in paraffin wax. Sections (3–5 µm thick) were prepared and stained with hematoxylin and eosin (H&E) using standard protocols. Tissue sections were assessed as described previously (21, 50). In brief, each gut section preparation was digitally imaged at high resolution. Images were then analyzed using Aperio Image Scope-pathology Slide Viewing Software (Version 12.4.6. Leica Biosystems). For each section, 10 villi and crypts were selected at random, and data were presented as villi height, villi width, and crypt depth (all in µm). Combined data included at least three chickens per treatment group and included three sections per tissue and 10 measurements for each section.

### Isolation of RNA from tissue

Cecal tonsils were removed from each bird into RNA*later* at postmortem and then stored at −80°C. Total RNA was extracted from cecal tonsils using the Qiagen RNeasy mini kit (Qiagen, Crawley, UK) as per the manufacturer’s instructions. Approximately 30 mg of tissue was placed in 600 µL RNA lysis Buffer in Lysing Matrix D tubes. Cecal tonsils were disrupted in the FastPrep FP120 (Thermo Scientific) at 4 m/second for 1 minute. Lysate was transferred to a fresh microcentrifuge tube and centrifuged to remove undisrupted tissue before being mixed with 70% ethanol. Samples were then added to an RNeasy spin column and washed several times with proprietary Buffer RW1 and RPE. Then, RNA was eluted using 50 µL nuclease-free water and quantified using a Nanodrop One (Thermo Scientific). Purified total RNA was stored at −80°C.

### cDNA synthesis

One microgram of total RNA was converted to cDNA using the Bio-Rad iScript cDNA Synthesis Kit as per the manufacturer’s instructions. Chicken universal reference total RNA was used as a positive control (amsbio, Abingdon, UK). The conversion was performed on the Agilent Technologies AriaMx Real-Time PCR System (Agilent Technologies, Cheshire, UK) under the following conditions: reverse transcriptase priming (5 minutes, 25°C), reverse transcription (20 minutes, 46°C), and reverse transcriptase inactivation (1 minute, 95°C). Converted cDNA was stored at −20°C.

### Quantitative reverse transcriptase PCR (qRT-PCR)

Primer and probe sequences for eight target sequences were obtained from a previous publication (51). New primers were designed using Primer3 (https://primer3.ut.ee/) using nucleotide sequences for *Gallus gallus domesticus* deposited on The National Center for Biotechnology Information (NCBI; National Library of Medicine [NIH]). Primers and probes were manufactured by Eurofins Genomics (Table S5). Primers and probes were reconstituted to 100 µM with nuclease-free water and stored at −20°C. Probes were kept wrapped in aluminum foil to prevent light degradation of fluorophores. Primers and probes were diluted 10-fold prior to qRT-PCR. In a 96-well plate, the following was added to each well: 12.5 µL Agilent Technologies Brilliant II QPCR Master Mix (Agilent Technologies), 0.625 µL Forward/Reverse Primer and Probe, and 8.625 µL nuclease-free water to a final volume of 23 µL. To sample wells, 2 µL of each cDNA was added, to positive control wells, 2 µL of reverse transcribed Chicken Universal Reference total RNA (amsbio, Abingdon, UK) was added, and to negative control wells, 2 µL nuclease-free water was added. All reactions were performed in duplicate. Plates were placed in the AriaMx Real-Time PCR System, and the qRT-PCR was run using the following conditions: DNA polymerase activation (10 minutes, 95°C), 40 cycles of DNA denaturation (30 seconds, 95°C), and annealing (1 minute; Table S4). The AriaMx software calculated Cq values, and they were used to establish gene expression using the Pfaffl Method (52). In brief, average Cq values were calculated using Agilent Aria 1.8 Software (Agilent Technologies) before being used in the following equation, where GOI is the gene of interest and HKG is the house-keeping gene:


$$Gene\;Expression\;Ratio=\frac{{\left(Primer\;Efficiency\;GOI\right)}^{\Delta CtGOI}}{{\left(Primer\;Efficiency\;HKG\right)}^{\Delta CtHKG}}.$$

### Serum Amyloid A ELISA

The concentration of serum amyloid A (SAA) in chicken sera was determined using the Chicken SAA ELISA (Life Diagnostics, Inc., Pennsylvania, USA) as per the manufacturer’s instructions. Briefly, 100 µL of serum was added to a microcentrifuge tube and incubated in a 60°C water bath for 1 hour to denature interfering factors. Then, samples were diluted 10-fold in diluent (YD50-1). Then, 100 µL of standards (0–15 ng/mL) and samples were added to corresponding wells in duplicate. To this, 100 µL of horseradish peroxidase (HRP) conjugate was added, and the plate was incubated for 20 minutes at 25°C with shaking at 150 rpm. Plates were washed five times with wash solution before 100 µL of TMB (TMB11-1) was added to each well, and the plate was incubated for 20 minutes at 25°C with shaking at 150 rpm. The reaction was stopped with 100 µL Stop Solution, and absorbance was recorded at 450 nm on a BMG Labtech FLUOstar Omega.

### Correlation and linear discriminant analysis

The relationship between *Campylobacter* viable counts in the different body sites (ileum and cecum), the immunological status as measured levels of cytokines (IL-22, IL-17a, TGFβ, IFNγ, CXCLi1, and CXCLi2), and the occurrence of extraintestinal spread to the spleen was investigated using correlation and discriminant analysis. Log-transformed counts were used. The correlation analysis was used to assess the association between viable cell counts and cytokine levels. Discriminant analysis was used to assess the extent to which an extra-intestinal spread event to the spleen could be predicted based on the cell counts in the intestine and the measured cytokine levels. The greedy Wilkes function in the klaR package in R was used to assess the extent to which non-event and invasion events could be separated using a stepwise procedure, with a variable included in the discriminant analysis if it led to a significant increase in discrimination. This analytical approach was used because it specifically includes covariation between potential predictors in assessing separation. This obviates the need for complex interaction terms that would otherwise be required in a logistic regression format (with many potential covariate combinations).

### Statistical analysis

Graphical data and statistical analysis were performed using GraphPad Prism 8. All data were subjected to normality testing, with normally distributed data subjected to a two-way ANOVA. Data that did not meet the requirements of normality were subjected to Kruskal-Wallis tests. Differences between control and infection groups in *Campylobacter* load, cytokine expression, SAA, or gut architecture parameters (crypt depth, villus height, and villus width) were considered significant if *P* < 0.05.

## RESULTS

### *Campylobacter* strains of the chicken-specialist ST353 and ST464 lineage exhibit high-level colonization of chicken gut tissues

Experiment 1 was an industrial-scale *Campylobacter* infection trial consisting of two replicates, 11 treatment groups with eight *C. jejuni* strains from four lineages (Table S1). Data were analyzed by strain and by lineage. Individual strains were administered to Ross 308 chickens at 21 days of age and tested for *C. jejuni* in cecal (Fig. S2 and S3) and ileal (Fig. S4 and S5) contents at 7 and 14 dpi. All strains showed similar levels of cecal colonization at both time points in both replicates with the exception of LE127, which was significantly decreased at 7 dpi compared to the mixed *C. jejuni* infection and LE17. In the ileum, M1, NCTC11168, and LE127 showed decreased colonization at 7 dpi compared to other strains. A low level of cecal and ileal colonization was detected in the control birds of replicate 2 possibly due to contamination of quantification plates. Routine bird monitoring determined growth performance, mortality, hock burn, and footpad lesions (Table S5). In brief, average daily feed intake was significantly higher in M1-treated birds than in uninfected controls between 7 and 14 days post-infection. Average daily weight gain was significantly increased between birds receiving the mixed infection (LE17/LE104) compared with either LE104, LE55, or LE127 treated birds between 7 and 14 days post-infection. Overall mortality was 3.2%, but there were no significant differences in mortality incidence between any group. Hock burn scores associated with greater tissue damage were significantly higher in LE104, LE89, LE34, and NCTC11168 compared to uninfected birds after 35 days of the experiment, but no differences were detected in footpad scores. This data confirmed that gut colonization and clinical data were strongly strain dependent.

**Fig 1:**
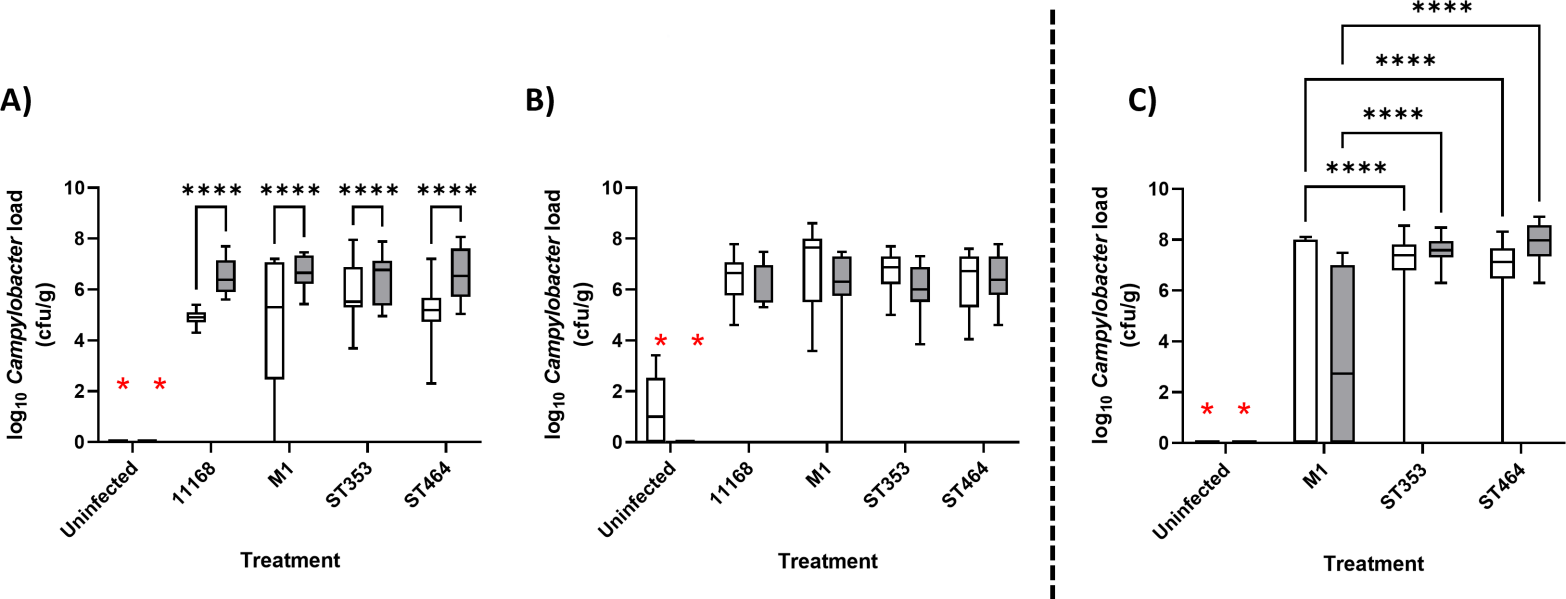
*Campylobacter* cecal load in large- and small-scale chicken infection trials Ross 308 broilers was challenged with ~10^5^ cfu *C*. *jejuni* (Fig. 9A; Table 1) by oral gavage with the uninfected control group given distilled water. At 7 (white) and 14 (gray) dpi, 10 birds in each treatment were randomly selected and humanely killed, and a fecal sample from the cecum was taken for enumeration of *Campylobacter* following serial dilution and plating. Data are presented as a box and whisker plot where the box defines the median and upper/lower interquartile values, and the whiskers confirm the range. Data values represent log *Campylobacter* counts in cecal samples. (**A**) Large-scale trial replicate 1. (**B**) Large-scale trial replicate 2. Large-scale trial plots include four strains in ST353 and 3 strains in ST464. (**C**)Small-scale trial where the plot includes two strains in ST353 and two strains in ST464. Significant differences between groups were calculated using two-way ANOVA and Tukey’s post hoc test. A *P* < 0.05 was considered significant. Symbols correspond to ****P < 0.0001. A red * denotes that all infection groups were significantly different to uninfected group.

**Fig 2:**
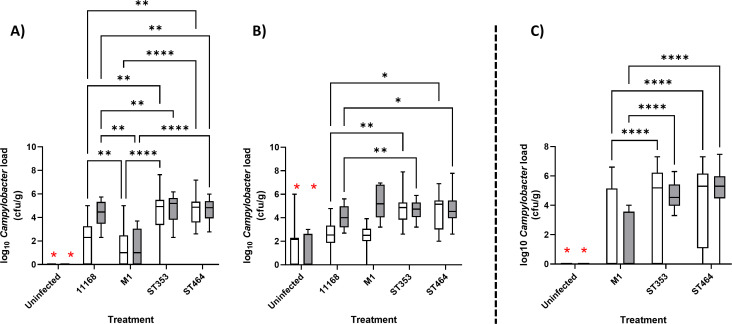
*Campylobacter* ileal load in large- and small-scale chicken infection trials Ross 308 broilers was challenged with ~10^5^ cfu *C*. *jejuni* (Fig. 9A; Table 1) by oral gavage with the uninfected control group given distilled water. At 7 (white) and 14 (gray) dpi, 10 birds in each treatment were randomly selected and humanely killed, and a fecal sample from the ileum was taken for enumeration of *Campylobacter* following serial dilution and plating. Data are presented as a box and whisker plot where the box defines the median and upper/lower interquartile values, and the whiskers confirm the range. Data values represent log *Campylobacter* counts in cecal samples. (**A**) Large-scale trial replicate 1. (**B**) Large-scale trial replicate 2. Large-scale trial plots include four strains in ST353 and 3 strains in ST464. (**C**) Small-scale trial where the plot includes two strains in ST353 and two strains in ST464. Significant differences between groups were calculated using two-way ANOVA and Tukey’s post hoc test. A *P* < 0.05 was considered significant. Symbols correspond to *P < 0.05, **P < 0.01, and ****P < 0.0001. A red * denotes that all infection groups were significantly different to uninfected groups.

Lineage-dependent colonization of each tissue was calculated by combining results for relevant *C. jejuni* strains, and the data presented by sequence type (Fig. 1). *C. jejuni* cecal load was significantly higher for the *C. jejuni*-infected chicken groups as compared to uninfected controls for both replicates (Fig. 1A and B, red stars). There were no significant differences identified between the standard test strains, M1 and NCTC11168, compared to the ST353 or ST464 treated chickens in either replicate of Experiment 1, although a time-dependent increase in cecal load was observed in the first experimental replicate (Fig. 1A). The *Campylobacter* ileal load was significantly increased for all C. *jejuni*-treated chickens compared to uninfected controls but was ~2 log lower than observed for cecal contents (Fig. 2A and B, red stars). At 7 dpi, *Campylobacter* ST353 and ST464 strains had significantly higher ileal loads as compared to *Campylobacter* M1 or NCTC11168. At 14 dpi, the level of ST353 and ST464 strains remained significantly higher than *Campylobacter* NCTC11168 (both replicates) and M1 (first replicate), respectively.

To extend and confirm these findings, a second independent, small-scale experiment (Fig. 1C and 2C; Fig. S8 and S9) was carried out at a different institution (Boxmeer). Experiment 2 included six groups: an uninfected control (U), *Campylobacter* M1, two ST353 strains (LE17 and LE55), and two ST464 strains (LE104 and LE142). Significant increases in cecal and ileal load were detected as compared to uninfected controls (Fig. 1C and 2C, red stars). Furthermore, at both 7 and 14 dpi, *Campylobacter* ST353 and ST464 strains had significantly higher loads in the cecum and ileum as compared to *Campylobacter* M1 (Fig. 1C and 2C). These findings demonstrate that *Campylobacter* strains from the ST353 and ST464 chicken-specialist lineages show strong robust and reproducible colonization of cecal and ileal contents in large- and small-scale chicken infection trials.

### *Campylobacter* strains belonging to ST353 and ST464 can be detected in extraintestinal tissues, with significant increases in splenic load

**Fig 3:**
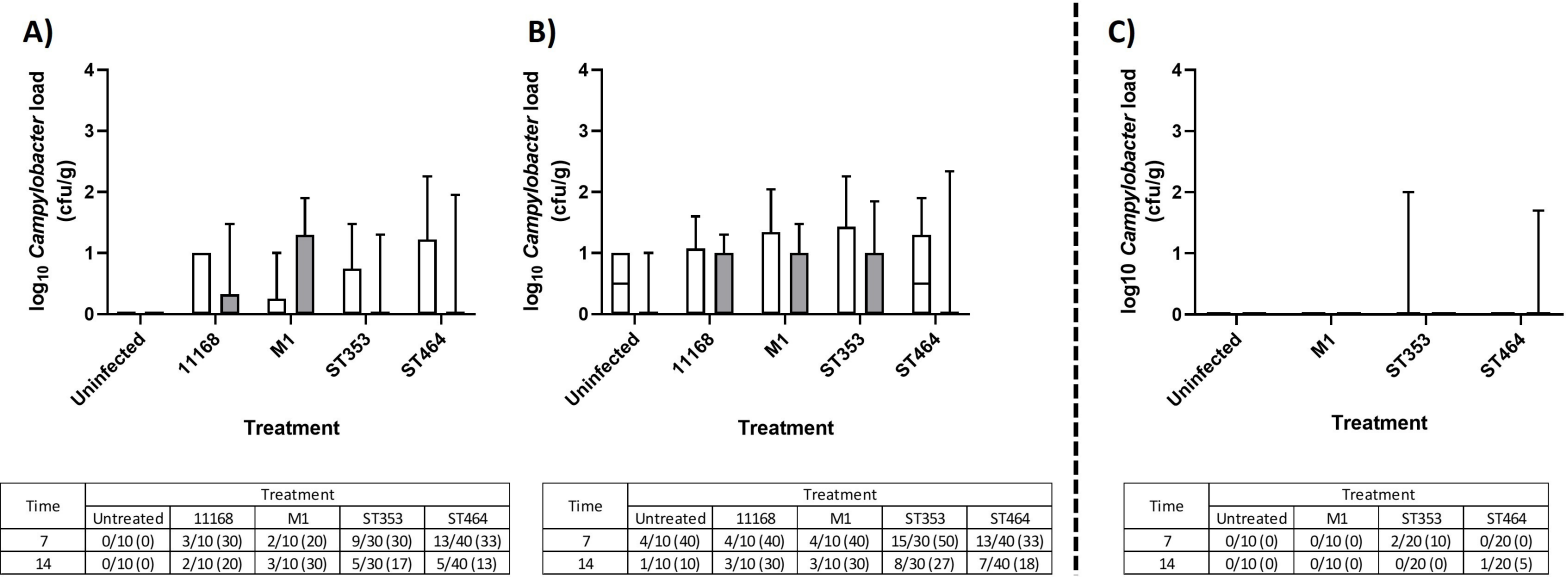
*Campylobacter* liver load in large- and small-scale chicken infection trials Ross 308 broilers was challenged with ~10^5^ cfu *C*. *jejuni* (Fig. 9A; Table 1) by oral gavage with the uninfected control group given distilled water. At 7 (white) and 14 (gray) dpi, 10 birds in each treatment were randomly selected and humanely killed, and a sample of liver tissue was taken and homogenized prior to enumeration of *Campylobacter* following serial dilution and plating. Data are presented as a box and whisker plot where the box defines the median and upper/lower interquartile values, and the whiskers confirm the range. Data values represent log *Campylobacter* counts in cecal samples. Tables represent a count of *Campylobacter*-positive chickens calculated from “direct counts” with percentages shown in brackets. (A) Large-scale trial replicate 1. (B) Large-scale trial replicate 2. Large-scale trial plots include four strains in ST353 and three strains in ST464. (C) Small-scale trial where the plot includes two strains in ST353 and 2 strains in ST464. Significant differences between groups were calculated using two-way ANOVA and Tukey’s post hoc test. A *P* < 0.05 was considered significant. No significant differences were detected.

**Fig 4:**
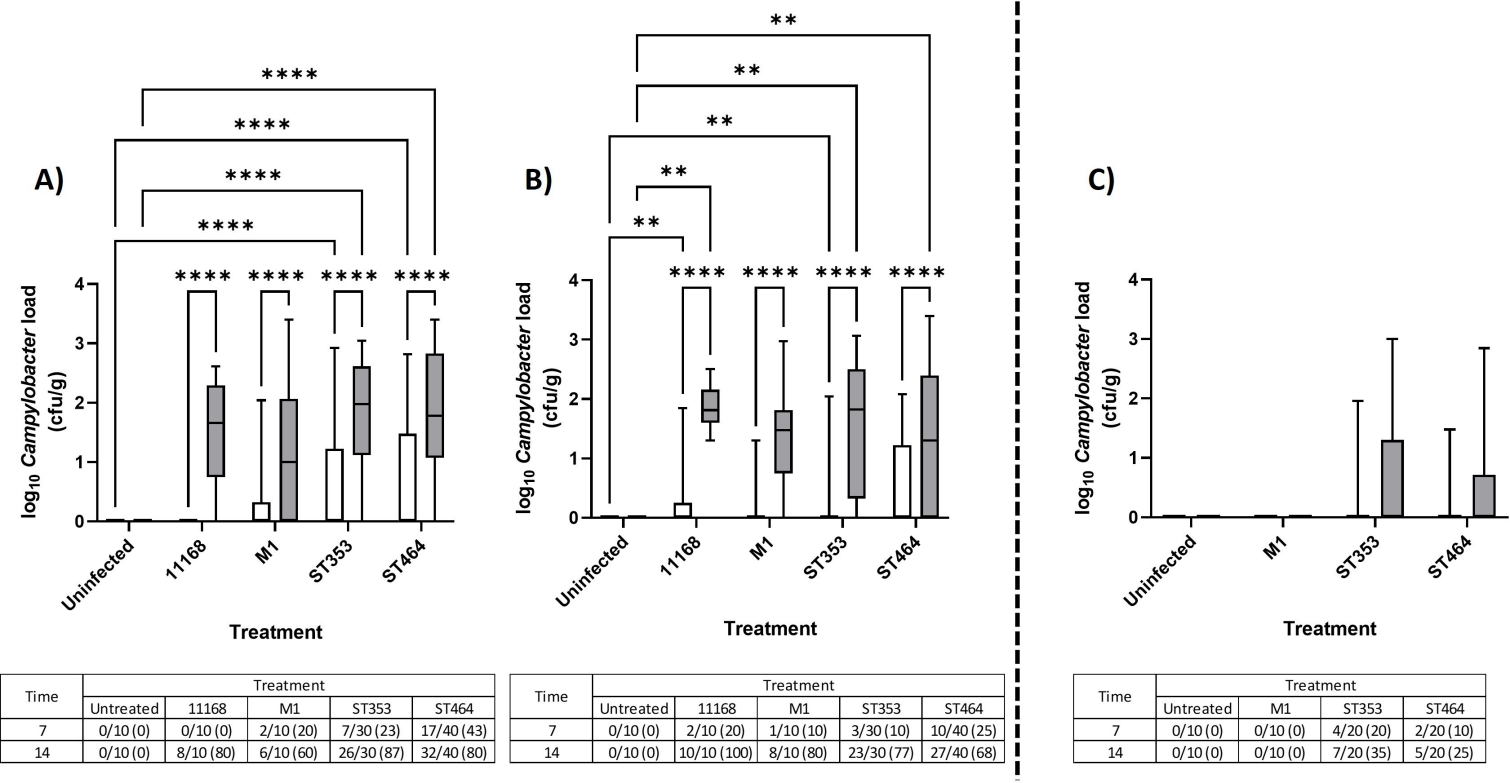
*Campylobacter* spleen load in large- and small-scale chicken infection trials Ross 308 broilers was challenged with ~10^5^ cfu *C*. *jejuni* (Fig. 9A; Table 1) by oral gavage with the uninfected control group given distilled water. At 7 (white) and 14 (gray) dpi, 10 birds in each treatment were randomly selected and humanely killed, and a sample of spleen tissue was taken and homogenized prior to enumeration of *Campylobacter* following serial dilution and plating. Data are presented as a box and whisker plot where the box defines the median and upper/lower interquartile values, and the whiskers confirm the range. Data values represent log *Campylobacter* counts in cecal samples. Tables represent a count of *Campylobacter*-positive chickens calculated from “direct counts” with percentages shown in brackets. (A) Large-scale trial replicate 1. (B) Large-scale trial replicate 2. Large-scale trial plots include four strains in ST353 and three strains in ST464. (C) Small-scale trial where the plot includes two strains in ST353 and two strains in ST464. Significant differences between groups were calculated using two-way ANOVA and Tukey’s post hoc test. A *P* < 0.05 was considered significant. Symbols correspond to **P < 0.01 and ****P < 0.0001.

To test for extraintestinal spread, homogenates of two internal organs were examined. Detection of *Campylobacter* in both liver (Fig. 3; Fig. S6 and S10; Table S6) and spleen (Fig. 4; Fig. S7 and S11; Table S7) homogenates confirmed that all of these strains were capable of spreading to extraintestinal tissues. In both replicates of Experiment 1, *Campylobacter* was detected at low levels in up to 33% and 50% of chicken livers, respectively (Fig. 3A and B sub-tables) at loads that were not significant relative to the control group (Fig. 3A and B). In Experiment 2, only *Campylobacter* ST353 (10%) and ST464 (5%) were detected in a small subset of livers, whereas M1 was undetectable (Fig. 3C and sub-table). Strikingly, Experiment 1 showed a time-dependent increase in *Campylobacter* splenic load for all strains to an average level of >10 cfu/g with these loads being significantly higher for the ST353 and ST464 strains than uninfected controls (Fig. 4A and B). Splenic colonization was also detected for ST353 and ST464 strains in Experiment 2 at similar loads (Fig. 4C). In summary, the *Campylobacter* ST353 and ST464 lineages show strong, robust, and reproducible extraintestinal spread to the spleen and sporadic, moderate-level spread to the liver. While NCTC11168 and M1 showed similar trends, they did not reach significance.

### *Campylobacter* sequence type-dependent induction of cecal tonsil-derived cytokines

**Fig 5:**
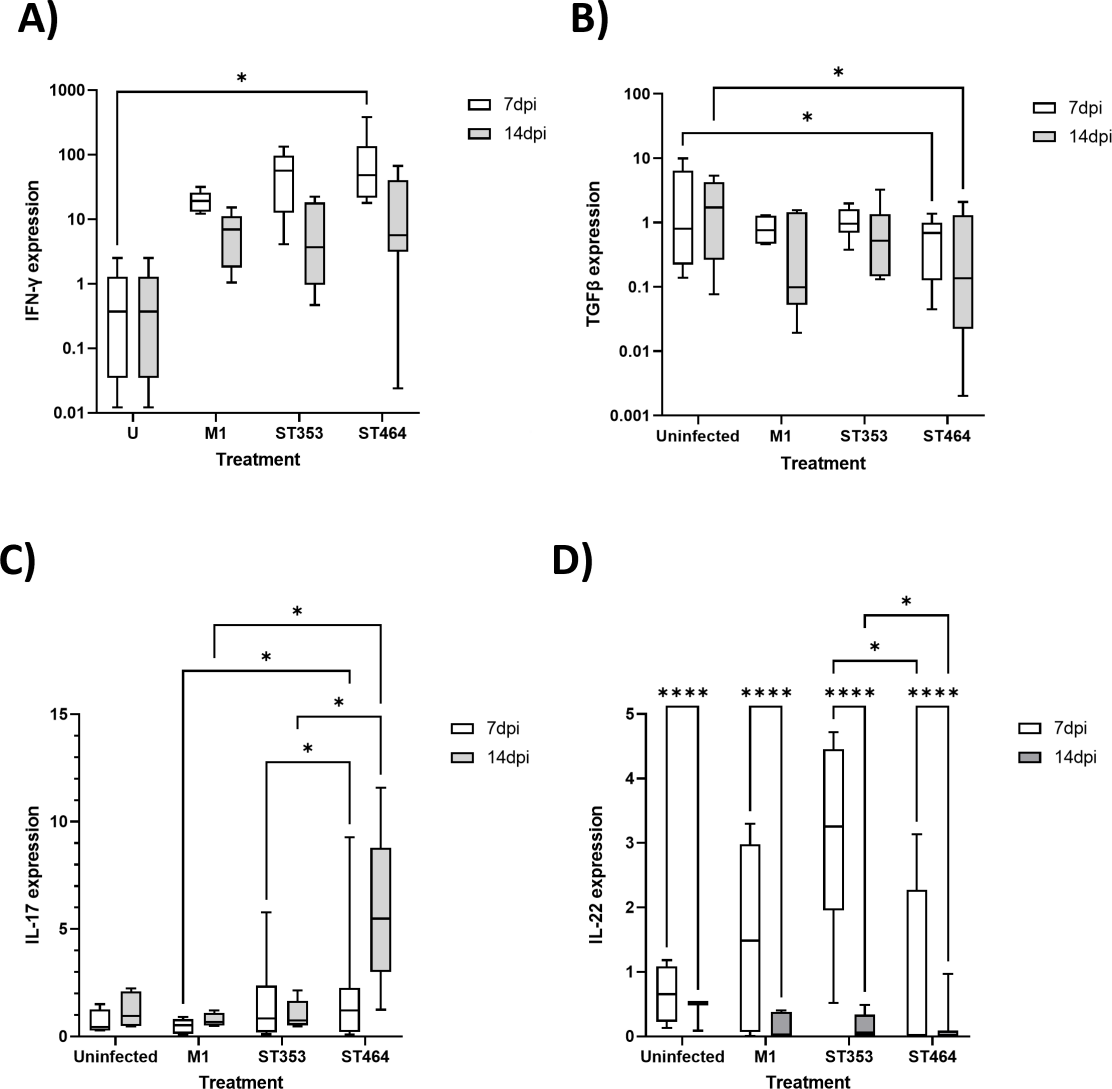
Cecal tonsil levels of IFNγ and TGFβ in large-scale chicken infection trials Ross 308 broilers were challenged with ~10^5^ cfu *C*. *jejuni* (Fig. 9A; Table 1) by oral gavage with the uninfected control group given distilled water. At 7 and 14 dpi, 10 birds in each treatment were randomly selected and humanely killed, and a sample of cecal tonsil was taken and stored in RNAlater prior to RNA isolation and PCR analysis of IFNγ and TGFβ transcripts. Data are presented as a box and whisker plot where the box defines the median and upper/lower interquartile values, and the whiskers confirm the range. Data values represent log-transformed gene expression using the Pfaffl method. Samples were used from the first large-scale trial only, and up to six chickens were used for each time point. (**A**) IFNγ gene expression. (**B**) TGFβ gene expression. (**C**) IL-17 gene expression. (**D**) IL-22 gene expression. Plots include an uninfected control and the M1 positive control. Then, data from two strains (LE17 and LE55) were combined for ST353, and two strains (LE104 and LE142) were combined for ST464. Significant differences between groups were calculated using two-way ANOVA and Tukey’s post hoc test. A *P* < 0.05 was considered significant. Symbols correspond to *P < 0.05.

Local immune responses in gut tissues were assessed by measuring cytokine cDNA levels in cecal tonsils from replicate 1 of Experiment 1 (Fig. 5; Fig. S12). The level of the Th1 cytokine IFNγ was increased in response to *Campylobacter* infection at both 7 and 14 dpi but only reached significance at 7 dpi (vs uninfected control) for ST464 strains (Fig. 5A). Consistent with this response, the Treg cytokine TGFβ was decreased at both 7 and 14 dpi but only reached significance for ST464 strains (Fig. 5B). The barrier-protective cytokines, IL-17A and IL-22, exhibited strain-specific induction kinetics (Fig. 5C and D). The ST464 strains induced significantly more IL-17 than either M1 or ST353 strains at both 7 and 14 dpi (Fig. 5C). In contrast, IL-22 was significantly reduced for all treatments, including the control, between 7 and 14 dpi. Furthermore, the ST464 strains had significantly reduced IL-22 expression compared to ST353 strains at both 7 and 14 dpi (Fig. 5D). Measurement of the chemokines, CXCLi1 and CXCLi2, as well as the Treg anti-inflammatory cytokine IL-10, could be detected in all treatments, but no significant differences were identified over the time course studied (Fig. S12). Measurement of SAA levels in blood (Fig. S13) showed a time-dependent increase over the course of the trial but no differences between treatment groups. These findings suggest that *Campylobacter* ST464 strains elicit a coordinated inflammatory phenotype localized to the gut lymphatics with concurrent induction of Th1/Th17 cytokines, reductions in the Treg response with no increase in SAA in blood.

### *Campylobacter* strains belonging to ST353 and ST464 cause specific damage in the ileal crypts

**Fig 6:**
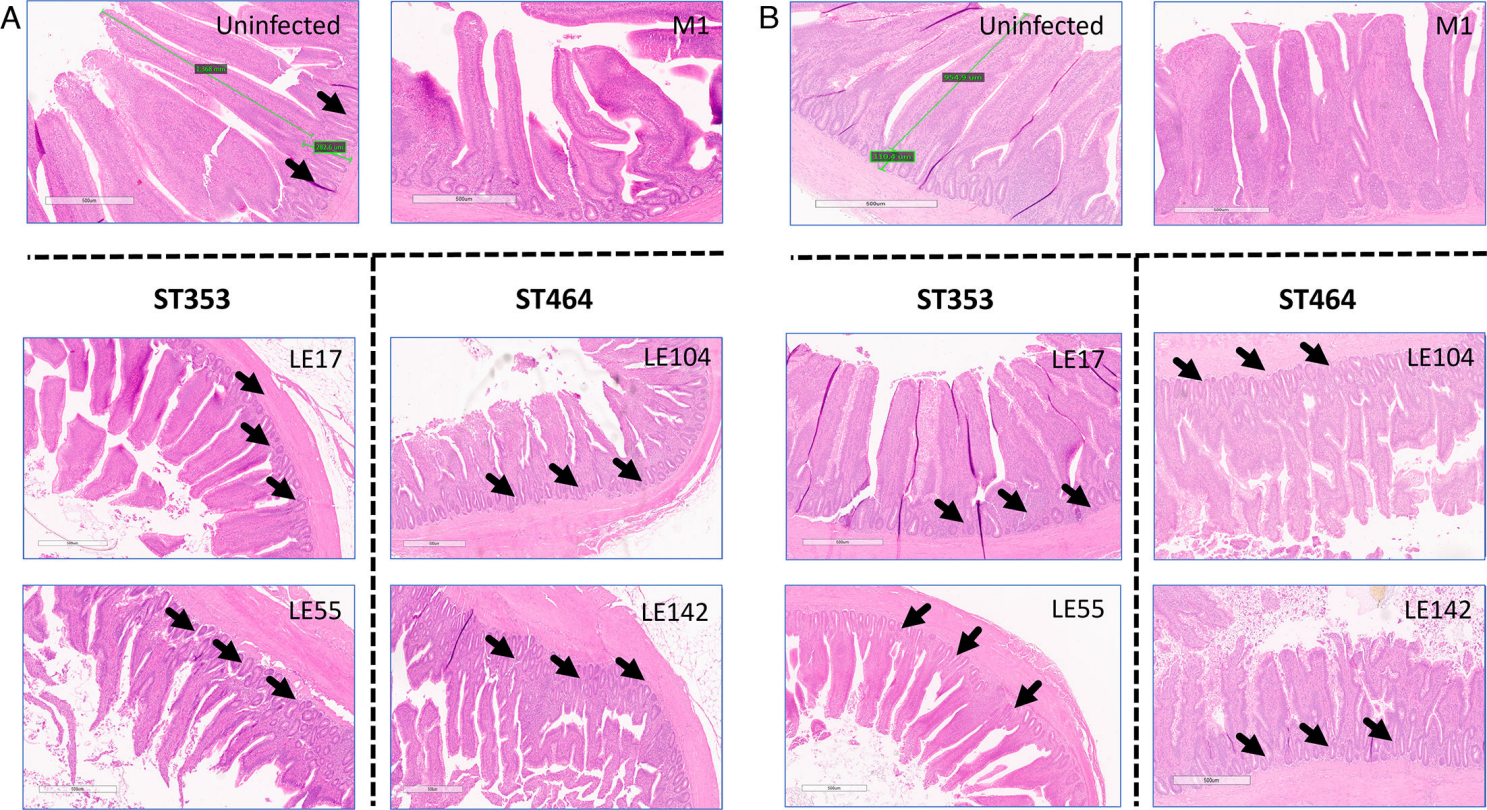
Chicken gut architecture from ileal tissue in large-scale chicken trial Ross 308 broilers was challenged with ~10^5^ cfu *C*. *jejuni* (Fig. 9A; Table 1) by oral gavage with the uninfected control group given distilled water. At 7 and 14 dpi, 10 birds in each treatment were randomly selected and humanely killed, and a tissue sample of ileum was taken and placed fixed in 4% (wt/vol) paraformaldehyde in PBS and routinely embedded in paraffin wax. Sections (3–5 µm thick) were prepared and stained with H&E using standard protocols. In brief, each gut section preparation was digitally imaged at high resolution. Images were then analyzed using Aperio Image Scope-pathology Slide Viewing Software (Version 12.4.6. Leica Biosystems). (**A**) Representative images of ileal tissue at 7 dpi from uninfected, positive control, M1, and two strains from ST353 and ST464, respectively. (**B**) Representative images of ileal tissue at 14 dpi from uninfected, positive control, M1, and two strains from ST353 and ST464, respectively. Green lines were generated in Aperio software to represent measures of villus height and crypt depth. Black arrows point to decreased crypt depth and altered morphology.

**Fig 7:**
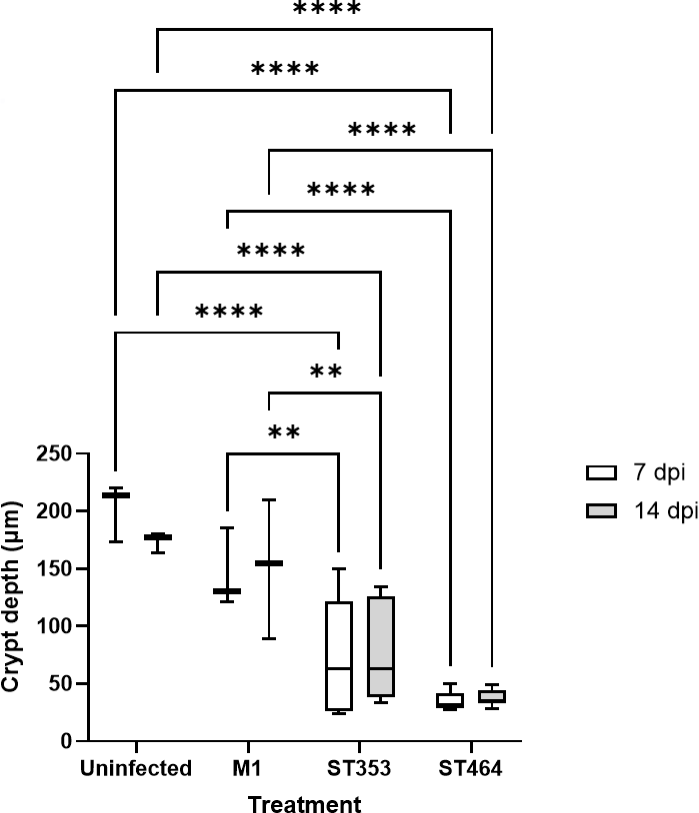
Quantification of ileal crypt depth in ileal tissue in large-scale chicken trial Ross 308 broilers was challenged with ~10^5^ cfu *C*. *jejuni* (Fig. 9A; Table 1) by oral gavage with the uninfected control group given distilled water. At 7 and 14 dpi, 10 birds in each treatment were randomly selected and humanely killed, and a tissue sample of ileum was taken and placed fixed in 4% (wt/vol) paraformaldehyde in PBS and routinely embedded in paraffin wax. Sections (3–5 µm thick) were prepared and stained with H&E using standard protocols. In brief, each gut section preparation was digitally imaged at high resolution. Images were then analyzed using Aperio Image Scope-pathology Slide Viewing Software (Version 12.4.6. Leica Biosystems). For each section, 10 villi and crypts were measured, and data were presented as crypt depth (µm). Combined data included at least three chickens per treatment group and included three sections per tissue and 10 measurements for each section. Significant differences between groups were calculated using two-way ANOVA and Tukey’s post hoc test. A *P* < 0.05 was considered significant. Symbols correspond to **P < 0.01 and ****P < 0.0001.

Tissue damage was assessed for Experiment 1. The intestinal architecture was imaged (Fig. 6A and B; Fig. S15A and B) and quantified (Fig. 7; Fig. S14 and S16) by measuring villus height, villus width, and crypt depth in ileum and cecum. In the ileum, significant decreases in crypt depth were observed between uninfected and M1-, ST353-, and ST464-infected chickens at both time points (Fig. 6A, B and 7). There were, however, no differences in ileal villus height or width (Fig. S14) or for the villi in cecal tissues (Fig. S15A, B and S16). These findings suggest that *C. jejuni* strains can specifically damage ileal intestinal crypts with the severity being strain dependent and increasing from low damage with M1, intermediate with ST353 strains and high damage with ST464 strains.

### Discriminant analysis of *Campylobacter* cell counts and immune parameters suggest host-pathogen interdependencies in the chicken gut

**Fig 8:**
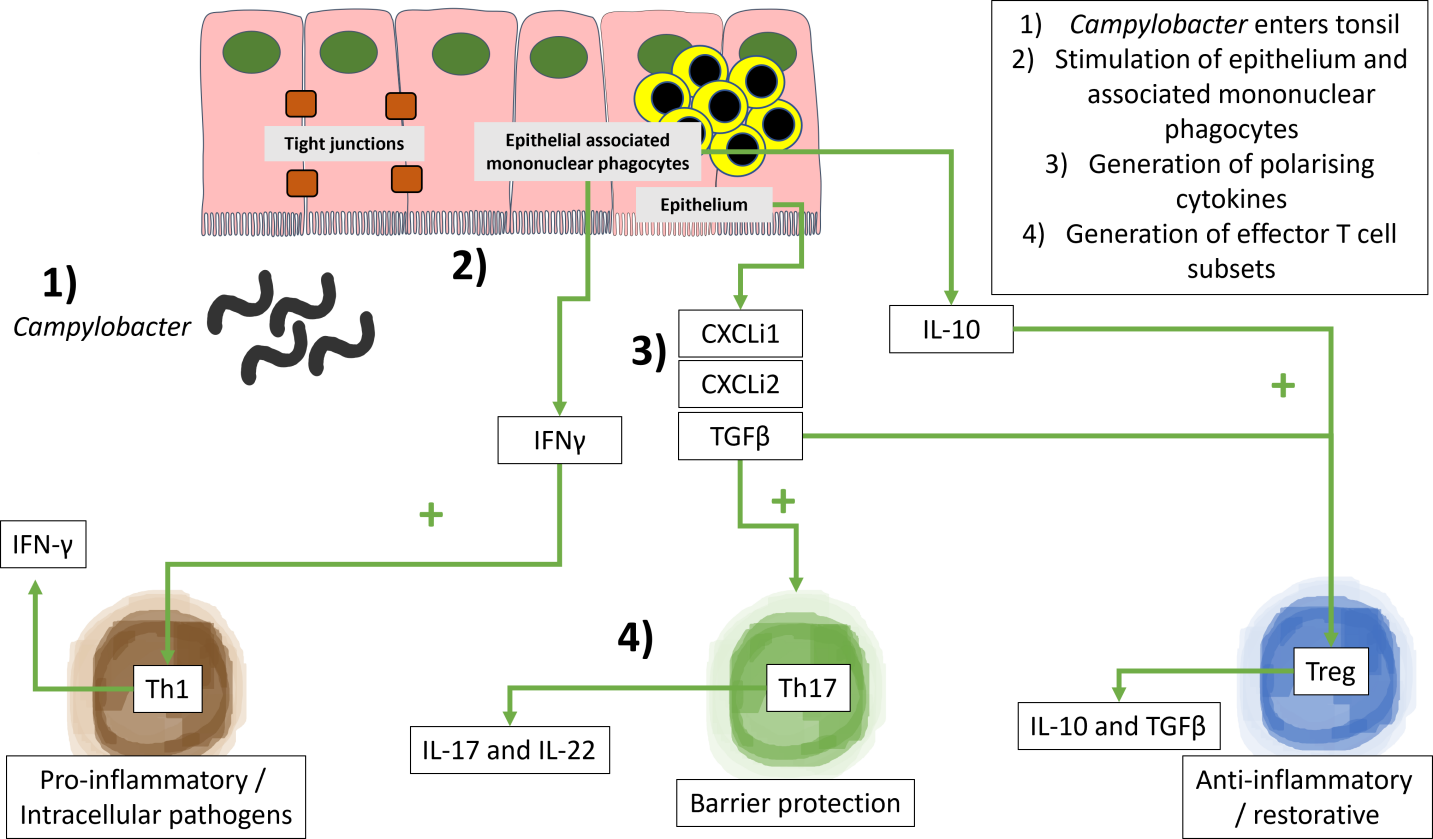
*Campylobacter*-induced cytokines in the chicken cecal tonsil *Campylobacter* and its antigens arrive in the cecal tonsil (1) and stimulate the epithelium and epithelial associated mononuclear cells (2) to generate polarizing cytokine (3) and effector T cell subsets (4). The current analysis has assumed a conventional structure of the cecal tonsil, which includes a specialized lymphoepithelium, a sub-epithelial zone, germinal centers, and interfollicular areas and mononuclear phagocytes residing below the epithelium. Therefore, CXCLi1/2 and TGFβ are produced by the epithelium. IFNγ, IL-10, and TGFβ are produced by mononuclear phagocytes. The combination of IFNγ and TGFβ provides an immune environment conducive to the development of Th1, Th17, and Treg helper lymphocytes.

Associations between *Campylobacter* counts in gut tissues, extraintestinal tissues, and cecal tonsil-derived cytokines were assessed using correlation and LDA. These were to confirm if immunological responses in gut tissues could predict extraintestinal spread. It was reasoned that *Campylobacter* and its antigens arrive in the cecal tonsil and stimulate cytokine production. For this analysis, a conventional structure of the cecal tonsil was assumed (Fig. 8), with a specialized lymphoepithelium, a sub-epithelial zone, germinal centers, and interfollicular areas and mononuclear phagocytes residing below and between the epithelium (53). A further assumption was that CXCLi1, CXCLi2, and TGFβ are produced by the epithelium, and IFNγ, IL-10, and TGFβ are produced by mononuclear phagocytes. The combination of IFNγ and TGFβ provides an immune environment conducive to the development of Th1, Th17, and Treg helper lymphocytes (Fig. 8).

**Fig 9:**
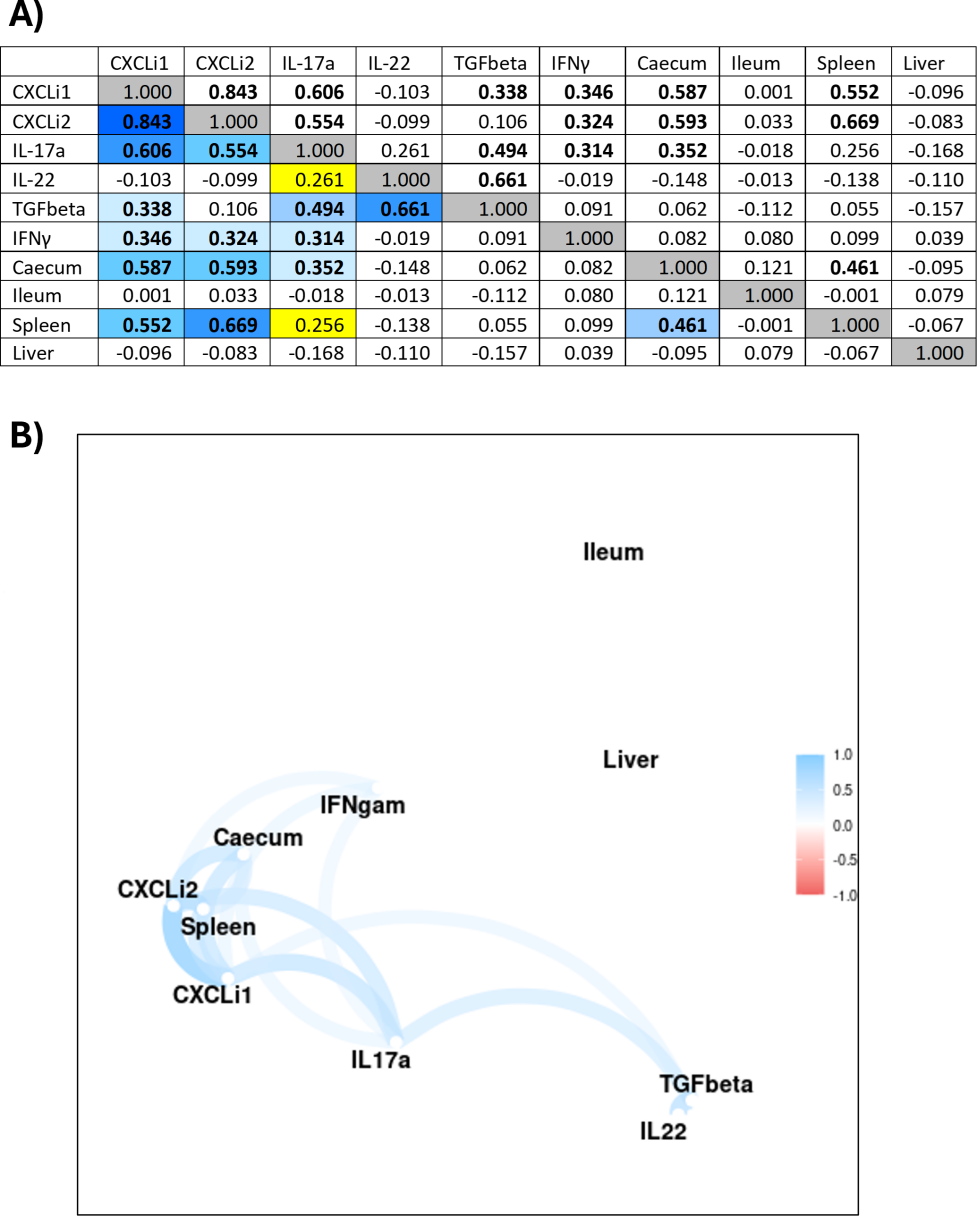
Correlation network of associations between immune parameters and *Campylobacter* load in tissues. (**A**) Correlation coefficients were derived using data from 52 chickens with no missing values. The critical value for the Pearson correlation coefficient at 50 degrees of freedom was 0.27. Bold values indicate significant correlations at *P* < 0.05. Blue cells indicate significant associations with the degree of blueness indicating the strength (this is consistent with the correlation network in panel B). Yellow cells indicate trending toward significance (*P* < 0.1). (**B**) Data from the pairwise correlation analysis was combined to form a visual representation of associations between parameters in a correlation network. Blue and red are positive and negative correlations, respectively.

Ten parameters were assessed for potential interdependencies: CXCLi1, CXCLi2, IL-17A, IL-22, TGFβ, IFNγ, and *Campylobacter* load in the cecum, ileum, liver, and spleen. The matrix of correlation coefficients comparing cytokine levels and *Campylobacter* load (Fig. 9) used data from a total of 52 chickens having the full complement of measurements. The critical value for the Pearson correlation coefficient at 50 degrees of freedom was 0.27, and all significant correlations are shown in bold. Strong correlations were observed between CXCLi1, CXCLi2, IFNγ, and IL-17A in any combination (Fig. 9). Levels of TGFβ and IL-22 were also positively associated. *Campylobacter* cell counts in the ileum and liver (which represent extraintestinal spread) were not significantly associated with cytokine levels or *Campylobacter* cell counts in the cecum (0.079 and −0.095, respectively). However, *Campylobacter* cell counts in the spleen (which also represents extraintestinal spread) were positively associated with cell counts in the cecum (Fig. 9, 0.461) and levels of CXCLi1 and CXCLi2. The association between the spleen and cecum with other parameters was visualized with a correlation network of significant correlations (Fig. 9). Furthermore, the results of a separate LDA showed that an invasion event in the spleen could be predicted with 71% accuracy from levels of CXCLi1, TGFβ, and IFNγ (Table 1).

**TABLE 1:** LDA of associations between immune parameters and splenic *Campylobacter* load^*a*^

Variable^*b*^	Wilks lambda	Overall *P* value^*c*^	*P* value diff^*c*^
IFNγ	0.92340	0.04709	0.04709
CXCLi1	0.77880	0.00218	0.00400
TGFβ	0.71130	0.00089	0.03790

^*a*^ Data generated in Experiment 1 for the cytokines, IFNγ, TGFβ, and CXCLi1 together with *Campylobacter* counts from the spleen were subjected to linear discriminant analysis.

^*b*^ Immune variables predicting spleen invasion.

^*c*^ The reliability of the parameter in predicting spleen invasion events. Predicted and observed values showed true positives at 67% and true negatives at 76%. The overall accuracy was 71%.

## DISCUSSION

*Campylobacter* strains from the generalist clonal complexes, CC21 (14, 32, 33) and CC45 (12, 32, 54), cause disease in chickens and can be transmitted through the food chain to humans (55–57). Identification of *Campylobacter* in chicken extraintestinal tissues (e.g., liver and muscle) suggests that survival and spread in internal tissues may be an important transmission route. To date, there is a paucity of experimental studies that address the potential of pathogenic chicken strains (36, 37, 39) and specialist lineages to cause extraintestinal spread. To address this knowledge gap, *Campylobacter* strains from two chicken-specialist lineages (ST353 and ST464) were obtained from the gut or liver tissues of naturally infected chickens (37, 38), and their infection biology was compared to two strains from generalist lineages.

Differential gut colonization of *Campylobacter* sequence types was detected in our chicken experiments. All strains colonized the cecum at relatively high and consistent loads, but colonization of ileum occurred earlier and at higher loads for ST353 and ST464 strains compared to the M1 and NCTC11168 strains. This is consistent with previous experimental infection work showing that *Campylobacter* strains have unique infection dynamics (14, 32, 39). This observation of chicken specialist sequence types ST353 (55) and ST464 (58) having faster infection kinetics than the generalist types (e.g., ST45 and ST21) may result from better adaption of specialist sequence types to the chicken gut as suggested previously (55, 57). In addition, recent studies have shown that *Campylobacter* ST353 isolates that were isolated on poultry farms showed associations with improved survival over other sequence types (59) and were associated with a winter peak in human gastroenteritis cases (60). Similarly, *Campylobacter* ST464 isolates have been found to express virulence factors with functions that enhance invasion and colonization of host tissues and also in immune evasion (61). Therefore, our observations of improved colonization are consistent with the limited data available on these chicken specialist sequence types.

One strength of our experimental design was the mimicking of normal commercial conditions with a high housing capacity and stocking density as found in the commercial, intensive poultry production model. Our two trials had similar terminal stocking densities of 33 kg/m^2^, but they differed in group size by 45–10 broiler chickens. Indeed, differences in extraintestinal spread between the two experiments may be attributable to the differences in group size (Fig. 3A, B, 4A, B vs 3C and 4C). Studies in chickens have found that increased group size and/or stocking density results in decreased physical performance (e.g., weight gain) (62–65), increased psychological and physiological stress, and decreased physical welfare (hock burn, breast blisters, and pododermatitis) (66, 67). Indeed, group size remains vitally important for social dynamics in natural populations of chickens (68). If, as our results suggest, there is a relationship between “increased group size and extraintestinal spread,” there are significant implications for the chicken industry where “group sizes” of up to 50,000 chickens are not unusual. Therefore, simple strategies to reduce group size (e.g., “biosecurity cubes”) into a set of sub-flocks may improve productivity and have the potential to upscale, thus putting the welfare of food-producing animals at a higher priority (69, 70). We recognize that increasing the space allocation per bird and reducing stocking density could influence parameters such as stress levels (71, 72), gut health and disease (66, 73, 74), welfare (12, 67, 75), and overall performance (76, 77). However, investigating the effect of reduced stocking density was beyond the scope of this study.

*Campylobacter* induced a significant, lineage-dependent cytokine polarization within the chicken cecal tonsil and histological changes in gut tissues which could contribute individually, additively, or synergistically to extraintestinal spread. The observation that *Campylobacter* ST464 induced IFNγ and IL-17 but reduced TGFβ is indicative of strong Th1 and Th17 responses and a bias away from a milieu of maintaining T-regulatory lymphocytes (Tregs). This cytokine profile is compatible with a pro-inflammatory environment within the cecal tonsil and is consistent with other experimental studies (78–80). Indeed, an excessive unregulated pro-inflammatory milieu has been associated with poor gut health leading to diarrhea, increased hock burn, and pododermatitis lesions (21). This study detected decreases in TGFβ and no change in IL-10 production which may have contributed to decreased Treg production in the cecal tonsil, increasing the inflammatory environment within the gut wall. This outcome would be consistent with our previous SEM implicating Th17 responses as important in protection against *Campylobacter* (44). This altered inflammatory environment may have also contributed to extraintestinal spread to the liver (13%–30% of chickens at 14 dpi in Experiment 1) and spleen (60%–100% of chickens at 14 dpi in Experiment 1). However, while IL-17 production is clearly important in the chicken gut, its local induction and regulation remain complex as Chagneau et al. found that spread to the liver is associated with a blunted IL-17 response in the gut and high systemic inflammation (79), whereas Connerton et al. showed a preference for induction of IL-17 in cecal, over ileal, tissues (80).

There is an intriguing observation in this study where *C. jejuni* ST464-treated chickens have decreased IL-22 in a background of high IL-17. Both IL-17 and IL-22 are considered barrier-protective cytokines. Therefore, we do not believe that IL-17 would be inhibiting IL-22 at the 14 dpi timepoint. However, there is some evidence to suggest that IL-17 and IL-22 are regulated differently during the inflammatory response and thus have different induction kinetics and functions. IL-17 has a largely pro-inflammatory role on epithelial cells, whereas IL-22 has a protective and regenerative effect on epithelial cells (reviewed in reference 81). Although we do not want to overinterpret, the low level of IL-22 and the decreased crypt depth in ST464 may be consistent with a reduced epithelial regeneration/production. Furthermore, this dysregulation of IL-17/IL-22 may have been caused by the virulence of *C. jejuni* (61). Indeed, ST464 strains express (i) *Cj1426c*, *kfiD*, *Cj1432c*, *Cj1440c*, and *glf*, which are all involved in capsule biosynthesis and transport; (ii) *fcl* (putative fucose synthase) which is involved in lipopolysaccharide (LPS) biosynthesis; and (iii) *pseE* which is involved in O-linked flagellar glycosylation.

To our knowledge, the histological examination showing that ST353 and ST464 infection causes specific distortion and atrophy in ileal crypts, but not villi structure or cecal tissues, is the first demonstration of gut damage by specialist *Campylobacter* strains. Other authors have shown that *Campylobacter* negatively affects gut barrier integrity with decreases in villus height and crypt depth (13, 82), increases in *Escherichia coli* translocation (34), and associations between *Campylobacter* translocation from the gut with villus blunting and shallower crypts (83). Critically, the very specific damage to the ileal crypt at 7 and 14 dpi may be a pre-requisite to the later damage of villi observed by Munoz and co-workers (84). If the intestinal crypt is the major site for stem cell regeneration, epithelial cell proliferation, and villi construction, we speculate that early crypt damage (7 and 14 dpi) could lead to significant dysregulation in local host defense. Indeed, this early tissue damage may also be the point where extraintestinal spread is initiated and may be the prelude to compromised villi production and systemic inflammation at later timepoints.

While we interpret decreased crypt depth as an indication of gut damage, our blood marker for the acute phase response, SAA, showed no significant increase. Additionally, we did not have sufficient blood samples to assay further mediators. We also saw no difference in villi height or width in the ileum and no differences in any of the parameters in the cecum. These results are consistent with the low and inconsistent colonization to the liver and thus a lack of a robust acute phase response. More generally, standardized biomarkers in the chicken remain elusive (85). Candidate markers for gut injury include (i) histological parameters; (ii) intestinal permeability methods using lactulose, mannitol, and rhamnose sugars (86); (iii) cytokines; (iv) markers of epithelial cell shedding; (v) markers of apoptosis; (vi) pathogen16S ribosomal RNA; (vii) short-chain fatty acids. Similarly, markers of systemic inflammation include (i) C reactive protein, LPS-binding protein, D-lactate, and the epithelial-derived enzyme diaminoxidase.

Our detection of *Campylobacter* in the liver (up to 50% of chickens) and high loads in spleens (up to 87% of chickens) for the ST353 and ST464 strains (in both large- and small-scale experiments) supports similar evidence of spread to internal organs in other chicken infection models with different strains (12, 13, 27, 35). There are two potential routes for extraintestinal spread – vascular and/or lymphatic. The significant IFNγ and IL-17 responses detected in cecal tonsils and low systemic inflammation suggest a lymphatic route. Conversely, the strong robust *Campylobacter* load detected in the spleen suggests a vascular route of transmission. The mammalian spleen has no direct lymphatic connection, but surprisingly, there is a physical link between the avian mesentery and lymphatics (87, 88). This is a potentially simple and logical explanation that requires further experimentation. These routes were further explored through modeling, and our pairwise correlation analysis confirmed strong interdependencies between cecal *Campylobacter* load, CXCLi1, CXCLi2, and splenic *Campylobacter* load. Further confirmation was identified in the LDA where three cecal tonsil cytokines (IFNγ, TGFβ, and CXCLi1) were predictive of splenic invasion events with an accuracy of 71%. Genetic analyses of the bacterial isolates from multiple chickens (in our companion paper) indicate that transfer between these sites is possible and, given the higher levels of colonization in the ceca as compared to the ileum, maybe the most likely source of spread (89).

While this study did not recapitulate the statistically significant associations between the ileum and liver shown previously with different *Campylobacter* strains (13, 31, 32), an intriguing association between cecum and spleen has been identified here. We speculate that cecal *Campylobacter* stimulates CXCLi1/2, a potent set of neutrophil chemokines and drivers of inflammation. In addition, cecal *Campylobacter* load also stimulates IFNγ and TGFβ, which in turn induce IL-17 and IL-22. Thus, a proinflammatory drive stimulated by *Campylobacter* in the cecum activates the cecal tonsil (increased IFNγ and IL-17A but decreased TGFβ), leading to spillover into the lymphatics, facilitating *Campylobacter* spread to the spleen. The use of correlation and LDA has proven a useful tool to predict invasive events (31, 44) and to discriminate bacterial pathogens (90) from cytokine responses and should be combined with further chicken trials to understand the mechanisms underlying extraintestinal spread.

Previous modeling work in our team has predicted that *Campylobacter* colonizes the chicken gut from the “upper gut” downward (ileum to the cecum) (31); however, this scenario does not explain the significantly higher loads in the cecum vs ileum (10^7^ cfu/g and 10^5^ cfu/g, respectively) in this study. Some drivers of this interesting phenotype are (i) immunoprotective antibodies in the ileum (31); (ii) the tolerogenic responses in the lower gut due to induction of IL-10 and the transcription factor LPS-induced TNFα factor (17, 35); and (iii) the differences in oxygen tension between lumen and epithelial crypts (16). In addition, the role of the ceca may be important here. The ceca are a pair of blind-ended pouches at the junction between ileum and colon. The ceca and ileal microbiota undergo a series of transitions as broilers mature until two unique complex communities are generated after 14 days of age (91). The mature ceca is a microaerobic to anoxic habitat (low to no oxygen). This environment favors the major roles of the ceca in carbohydrate digestion, nitrogen recycling, the colonization of gut anaerobes, and the modulation of gut microflora. Indeed, our team has previously hypothesized that the physical and biological properties of this environment allow *C. jejuni* to survive in mixed population biofilms evading the immune system and utilizing iron and sulfur uptake systems (92). Confirmation of these important observations requires further detailed *in vivo* time series trials to examine the local compartmentalization of gut immunity to *Campylobacter*.

Our study used commercial-scale stocking densities and sufficient power to detect differences between infected and uninfected controls and between lineages. The inclusion of two biological replicates addressed reproducibility, and both replicates were similar for most of the biological outputs. There were, however, limitations: (i) cecal tonsils were sampled as a known surrogate biomarker of chicken gut inflammation, but these tissues may not fully reflect inflammatory responses occurring in ileal and cecal tissue; (ii) some variation was observed both within groups of birds and between Experiments 1 and 2, but it is not possible to determine if this was due to minor differences in sampling processes.

In conclusion, this study has confirmed that *Campylobacter* ST353 and ST464, chicken “specialist” lineages, can colonize the chicken gut and can translocate to reach extraintestinal tissues such as the liver and spleen. Local immune responses in the cecal tonsils confirm an inflammatory milieu high in Th1 and Th17 markers and low in Treg factors. The greatest inflammation was generated by *C. jejuni* ST464 strains which also generated the most damage to intestinal crypts specifically in the ileum. Correlation and LDA identified a specific set of cytokine biomarkers (IFNγ, TGFβ, and CXCLi1) predictive of the risk of *Campylobacter* spleen invasion. This work confirms for the first time that the chicken-specialist lineages, ST353 and ST464, induce gut inflammation and damage crypt structures, leading to extraintestinal spread and increasing the risk of edible tissue contamination.

## SUPPLEMENTAL MATERIAL

Supplemental material aem.01614-24-s0001.docx Tables S1 to S7; Figures S1 to S16.

ASM does not own the copyrights to Supplemental Material that may be linked to, or accessed through, an article. The authors have granted ASM a non-exclusive, world-wide license to publish the Supplemental Material files. Please contact the corresponding author directly for reuse.

## References

[1] EFSA. 2022. Campylobacter. Available from: https://www.efsa.europa.eu/en/topics/topic/campylobacter

[2] Tam CC, O’Brien SJ. 2016. Economic cost of Campylobacter, norovirus and rotavirus disease in the United Kingdom. PLoS One 11:e0138526. doi:10.1371/journal.pone.0138526 26828435 PMC4735491

[3] Kaakoush NO, Castaño-Rodríguez N, Mitchell HM, Man SM. 2015. Global epidemiology of Campylobacter infection. Clin Microbiol Rev 28:687–720. doi:10.1128/CMR.00006-15 26062576 PMC4462680

[4] FSA. 2020. The burden of foodbourne disease in the UK 2018

[5] Lanier WA, Hale KR, Geissler AL, Dewey-Mattia D. 2018. Chicken liver-associated outbreaks of campylobacteriosis and salmonellosis, United States, 2000-2016: identifying opportunities for prevention. Foodborne Pathog Dis 15:726–733. doi:10.1089/fpd.2018.2489 30192164 PMC6247982

[6] Esan OB, Perera R, McCarthy N, Violato M, Fanshawe TR. 2020. Incidence, risk factors, and health service burden of sequelae of campylobacter and non-typhoidal salmonella infections in England, 2000-2015: a retrospective cohort study using linked electronic health records. J Infect 81:221–230. doi:10.1016/j.jinf.2020.05.027 32445725

[7] Hakeem MJ, Lu X. 2020. Survival and control of Campylobacter in poultry production environment. Front Cell Infect Microbiol 10:615049. doi:10.3389/fcimb.2020.615049 33585282 PMC7879573

[8] Jørgensen F, Sadler-Reeves L, Shore J, Aird H, Elviss N, Fox A, Kaye M, Willis C, Amar C, DE Pinna E, McLauchlin J. 2017. An assessment of the microbiological quality of lightly cooked food (including sous-vide) at the point of consumption in England. Epidemiol Infect 145:1500–1509. doi:10.1017/S0950268817000048 28236815 PMC9203327

[9] Cox NA, Hofacre CL, Bailey JS, Buhr RJ, Wilson JL, Hiett KL, Richardson LJ, Musgrove MT, Cosby DE, Tankson JD, Vizzier YL, Cray PF, Vaughn LE, Holt PS, Bourassaa DV. 2005. Presence of Campylobacter jejuni in various organs one hour, one day, and one week following oral or intracloacal inoculations of broiler chicks. Avian Dis 49:155–158. doi:10.1637/7234-070704R 15839431

[10] Cox NA, Richardson LJ, Buhr RJ, Fedorka-Cray PJ, Bailey JS, Wilson JL, Hiett KL. 2006. Natural presence of Campylobacter spp. in various internal organs of commercial broiler breeder hens. Avian Dis 50:450–453. doi:10.1637/7481-120205R.1 17039849

[11] Cox NA, Richardson LJ, Buhr RJ, Northcutt JK, Bailey JS, Cray PF, Hiett KL. 2007. Recovery of Campylobacter and Salmonella serovars from the spleen, liver and gallbladder, and ceca of six-and eight-week-old commercial broilers. J Appl Poult Res 16:477–480. doi:10.3382/japr.2006-00123

[12] Williams LK, Sait LC, Trantham EK, Cogan TA, Humphrey TJ. 2013. Campylobacter infection has different outcomes in fast- and slow-growing broiler chickens. Avian Dis 57:238–241. doi:10.1637/10442-110212-Reg.1 24689180

[13] Awad WA, Molnár A, Aschenbach JR, Ghareeb K, Khayal B, Hess C, Liebhart D, Dublecz K, Hess M. 2015. Campylobacter infection in chickens modulates the intestinal epithelial barrier function. Innate Immun 21:151–160. doi:10.1177/1753425914521648 24553586

[14] Humphrey S, Lacharme-Lora L, Chaloner G, Gibbs K, Humphrey T, Williams N, Wigley P. 2015. Heterogeneity in the infection biology of Campylobacter jejuni isolates in three infection models reveals an invasive and virulent phenotype in a ST21 isolate from poultry. PLoS One 10:e0141182. doi:10.1371/journal.pone.0141182 26496441 PMC4619714

[15] Shane SM. 1992. The significance of Campylobacter jejuni infection in poultry: a review. Avian Pathol 21:189–213. doi:10.1080/03079459208418836 18670933

[16] Beery JT, Hugdahl MB, Doyle MP. 1988. Colonization of gastrointestinal tracts of chicks by Campylobacter jejuni. Appl Environ Microbiol 54:2365–2370. doi:10.1128/aem.54.10.2365-2370.1988 3060015 PMC204261

[17] Hermans D, Pasmans F, Heyndrickx M, Van Immerseel F, Martel A, Van Deun K, Haesebrouck F. 2012. A tolerogenic mucosal immune response leads to persistent Campylobacter jejuni colonization in the chicken gut. Crit Rev Microbiol 38:17–29. doi:10.3109/1040841X.2011.615298 21995731

[18] Prescott JF, Bruin-Mosch CW. 1981. Carriage of Campylobacter jejuni in healthy and diarrheic animals. Am J Vet Res 42:164–165. 7224312

[19] Ruiz-Palacios GM, Escamilla E, Torres N. 1981. Experimental Campylobacter diarrhea in chickens. Infect Immun 34:250–255. doi:10.1128/iai.34.1.250-255.1981 7298187 PMC350849

[20] Sanyal SC, Islam KM, Neogy PK, Islam M, Speelman P, Huq MI. 1984. Campylobacter jejuni diarrhea model in infant chickens. Infect Immun 43:931–936. doi:10.1128/iai.43.3.931-936.1984 6698612 PMC264273

[21] Humphrey S, Chaloner G, Kemmett K, Davidson N, Williams N, Kipar A, Humphrey T, Wigley P. 2014. Campylobacter jejuni is not merely a commensal in commercial broiler chickens and affects bird welfare. MBio 5:e01364-14. doi:10.1128/mBio.01364-14 24987092 PMC4161246

[22] Cox NA, Richardson LJ, Buhr RJ, Bailey JS, Wilson JL, Hiett KL. 2006. Detection of Campylobacter jejuni in various lymphoid organs of broiler breeder hens after oral or intravaginal inoculation. Poult Sci 85:1378–1382. doi:10.1093/ps/85.8.1378 16903467

[23] Lopes GV, Ramires T, Kleinubing NR, Scheik LK, Fiorentini ÂM, Padilha da Silva W. 2021. Virulence factors of foodborne pathogen Campylobacter jejuni. Microb Pathog 161:105265. doi:10.1016/j.micpath.2021.105265 34699927

[24] Han Z, Willer T, Li L, Pielsticker C, Rychlik I, Velge P, Kaspers B, Rautenschlein S. 2017. Influence of the gut microbiota composition on Campylobacter jejuni colonization in chickens. Infect Immun 85:e00380-17. doi:10.1128/IAI.00380-17 28808158 PMC5649013

[25] Ijaz UZ, Sivaloganathan L, McKenna A, Richmond A, Kelly C, Linton M, Stratakos AC, Lavery U, Elmi A, Wren BW, Dorrell N, Corcionivoschi N, Gundogdu O. 2018. Comprehensive longitudinal microbiome analysis of the chicken cecum reveals a shift from competitive to environmental drivers and a window of opportunity for Campylobacter. Front Microbiol 9:2452. doi:10.3389/fmicb.2018.02452 30374341 PMC6196313

[26] Al Hakeem WG, Acevedo Villanueva KY, Selvaraj RK. 2023. The development of gut microbiota and its changes following C. jejuni infection in broilers. Vaccines (Basel) 11:595. doi:10.3390/vaccines11030595 36992178 PMC10056385

[27] Gharib NK, Rahimi S, Khaki P. 2012. Comparison of the effects of probiotic, organic acid and medicinal plant on Campylobacter jejuni challenged broiler chickens. J Agr Sci Tech 14:1485–1496.

[28] Awad WA, Aschenbach JR, Ghareeb K, Khayal B, Hess C, Hess M. 2014. Campylobacter jejuni influences the expression of nutrient transporter genes in the intestine of chickens. Vet Microbiol 172:195–201. doi:10.1016/j.vetmic.2014.04.001 24834798

[29] Newell DG, Fearnley C. 2003. Sources of Campylobacter colonization in broiler chickens. Appl Environ Microbiol 69:4343–4351. doi:10.1128/AEM.69.8.4343-4351.2003 12902214 PMC169125

[30] Bindari YR, Gerber PF. 2022. Centennial review: factors affecting the chicken gastrointestinal microbial composition and their association with gut health and productive performance. Poult Sci 101:101612. doi:10.1016/j.psj.2021.101612 34872745 PMC8713025

[31] Lacharme-Lora L, Chaloner G, Gilroy R, Humphrey S, Gibbs K, Jopson S, Wright E, Reid W, Ketley J, Humphrey T, Williams N, Rushton S, Wigley P. 2017. B lymphocytes play a limited role in clearance of Campylobacter jejuni from the chicken intestinal tract. Sci Rep 7:45090. doi:10.1038/srep45090 28332622 PMC5362810

[32] Chaloner G, Wigley P, Humphrey S, Kemmett K, Lacharme-Lora L, Humphrey T, Williams N. 2014. Dynamics of dual infection with Campylobacter jejuni strains in chickens reveals distinct strain-to-strain variation in infection ecology. Appl Environ Microbiol 80:6366–6372. doi:10.1128/AEM.01901-14 25107966 PMC4178652

[33] Sylte MJ, Shippy DC, Bearson BL, Bearson SMD. 2020. Detection of Campylobacter jejuni liver dissemination in experimentally colonized turkey poults. Poult Sci 99:4028–4033. doi:10.1016/j.psj.2020.03.042 32731990 PMC7597910

[34] Awad WA, Dublecz F, Hess C, Dublecz K, Khayal B, Aschenbach JR, Hess M. 2016. Campylobacter jejuni colonization promotes the translocation of Escherichia coli to extra-intestinal organs and disturbs the short-chain fatty acids profiles in the chicken gut. Poult Sci 95:2259–2265. doi:10.3382/ps/pew151 27143773

[35] Mortada M, Cosby DE, Akerele G, Ramadan N, Oxford J, Shanmugasundaram R, Ng TT, Selvaraj RK. 2021. Characterizing the immune response of chickens to Campylobacter jejuni (Strain A74C). PLoS One 16:e0247080. doi:10.1371/journal.pone.0247080 33720955 PMC7959354

[36] Stef L, Cean A, Vasile A, Julean C, Drinceanu D, Corcionivoschi N. 2013. Virulence characteristics of five new Campylobacter jejuni chicken isolates. Gut Pathog 5:41. doi:10.1186/1757-4749-5-41 24330718 PMC3866932

[37] John DA. 2019. Host pathogen responses of chicken cells in vitro and in vivo to a diverse population of Campylobacter strains PhD, Swansea

[38] Williams LK. 2024. Characterisation of Campylobacter from naturally infected chickens

[39] Pielsticker C, Glünder G, Aung YH, Rautenschlein S. 2016. Colonization pattern of C. jejuni isolates of human and avian origin and differences in the induction of immune responses in chicken. Vet Immunol Immunopathol 169:1–9. doi:10.1016/j.vetimm.2015.11.005 26827832

[40] Garcia JS, Byrd JA, Wong EA. 2018. Expression of nutrient transporters and host defense peptides in Campylobacter challenged broilers. Poult Sci 97:3671–3680. doi:10.3382/ps/pey228 29931274

[41] Nyati KK, Prasad KN, Kharwar NK, Soni P, Husain N, Agrawal V, Jain AK. 2012. Immunopathology and Th1/Th2 immune response of Campylobacter jejuni-induced paralysis resembling Guillain-Barré syndrome in chicken. Med Microbiol Immunol 201:177–187. doi:10.1007/s00430-011-0220-3 22102098

[42] Smith CK, Abuoun M, Cawthraw SA, Humphrey TJ, Rothwell L, Kaiser P, Barrow PA, Jones MA. 2008. Campylobacter colonization of the chicken induces a proinflammatory response in mucosal tissues. FEMS Immunol Med Microbiol 54:114–121. doi:10.1111/j.1574-695X.2008.00458.x 18647351

[43] John DA, Williams LK, Kanamarlapudi V, Humphrey TJ, Wilkinson TS. 2017. The bacterial species Campylobacter jejuni induce diverse innate immune responses in human and avian intestinal epithelial cells. Front Microbiol 8:1840. doi:10.3389/fmicb.2017.01840 29033908 PMC5626877

[44] Reid WDK, Close AJ, Humphrey S, Chaloner G, Lacharme-Lora L, Rothwell L, Kaiser P, Williams NJ, Humphrey TJ, Wigley P, Rushton SP. 2016. Cytokine responses in birds challenged with the human food-borne pathogen Campylobacter jejuni implies a Th17 response. R Soc Open Sci 3:150541. doi:10.1098/rsos.150541 27069644 PMC4821255

[45] Friis C, Wassenaar TM, Javed MA, Snipen L, Lagesen K, Hallin PF, Newell DG, Toszeghy M, Ridley A, Manning G, Ussery DW. 2010. Genomic characterization of Campylobacter jejuni strain M1. PLoS One 5:e12253. doi:10.1371/journal.pone.0012253 20865039 PMC2928727

[46] Parkhill J, Wren BW, Mungall K, Ketley JM, Churcher C, Basham D, Chillingworth T, Davies RM, Feltwell T, Holroyd S, Jagels K, Karlyshev AV, Moule S, Pallen MJ, Penn CW, Quail MA, Rajandream M-A, Rutherford KM, van Vliet AHM, Whitehead S, Barrell BG. 2000. The genome sequence of the food-borne pathogen Campylobacter jejuni reveals hypervariable sequences. Nature New Biol 403:665–668. doi:10.1038/35001088 10688204

[47] Skirrow MB. 1977. Campylobacter enteritis: a “new” disease. Br Med J 2:9–11. doi:10.1136/bmj.2.6078.9 871765 PMC1631297

[48] Mattick K, Durham K, Domingue G, Jørgensen F, Sen M, Schaffner DW, Humphrey T. 2003. The survival of foodborne pathogens during domestic washing-up and subsequent transfer onto washing-up sponges, kitchen surfaces and food. Int J Food Microbiol 85:213–226. doi:10.1016/s0168-1605(02)00510-x 12878380

[49] Dawkins MS, Donnelly CA, Jones TA. 2004. Chicken welfare is influenced more by housing conditions than by stocking density. Nature 427:342–344. doi:10.1038/nature02226 14737165

[50] Nguyen DTN. 2021. Relationship between the ratio of villous height:crypt depth and gut bacteria counts as well production parameters in broiler chickens. J Agric Dev 20:1–10. doi:10.52997/jad.1.03.2021

[51] Shini S, Kaiser P. 2009. Effects of stress, mimicked by administration of corticosterone in drinking water, on the expression of chicken cytokine and chemokine genes in lymphocytes. Stress 12:388–399. doi:10.1080/10253890802526894 19006006

[52] Pfaffl MW. 2001. A new mathematical model for relative quantification in real-time RT-PCR. Nucleic Acids Res 29:e45. doi:10.1093/nar/29.9.e45 11328886 PMC55695

[53] Olah I, Nagy N, Vervelde L. 2014. Structure of the avian lymphoid system, p 11–44. In Schat KA, Kaspers B, Kaiser P (ed), Avian immunology, 2nd ed

[54] Gracia MI, Sánchez J, Millán C, Casabuena Ó, Vesseur P, Martín Á, García-Peña FJ, Medel P. 2016. Effect of feed form and whole grain feeding on gastrointestinal weight and the prevalence of Campylobacter jejuni in broilers orally infected. PLoS One 11:e0160858. doi:10.1371/journal.pone.0160858 27500730 PMC4976972

[55] Sheppard SK, Cheng L, Méric G, de Haan CPA, Llarena A-K, Marttinen P, Vidal A, Ridley A, Clifton-Hadley F, Connor TR, Strachan NJC, Forbes K, Colles FM, Jolley KA, Bentley SD, Maiden MCJ, Hänninen M-L, Parkhill J, Hanage WP, Corander J. 2014. Cryptic ecology among host generalist Campylobacter jejuni in domestic animals. Mol Ecol 23:2442–2451. doi:10.1111/mec.12742 24689900 PMC4237157

[56] Gripp E, Hlahla D, Didelot X, Kops F, Maurischat S, Tedin K, Alter T, Ellerbroek L, Schreiber K, Schomburg D, Janssen T, Bartholomäus P, Hofreuter D, Woltemate S, Uhr M, Brenneke B, Grüning P, Gerlach G, Wieler L, Suerbaum S, Josenhans C. 2011. Closely related Campylobacter jejuni strains from different sources reveal a generalist rather than a specialist lifestyle. BMC Genomics 12:584. doi:10.1186/1471-2164-12-584 22122991 PMC3283744

[57] Sheppard SK, Colles F, Richardson J, Cody AJ, Elson R, Lawson A, Brick G, Meldrum R, Little CL, Owen RJ, Maiden MCJ, McCarthy ND. 2010. Host association of Campylobacter genotypes transcends geographic variation. Appl Environ Microbiol 76:5269–5277. doi:10.1128/AEM.00124-10 20525862 PMC2916502

[58] Cobo-Díaz JF, González del Río P, Álvarez-Ordóñez A. 2021. Whole resistome analysis in Campylobacter jejuni and C. coli genomes available in public repositories. Front Microbiol 12:662144. doi:10.3389/fmicb.2021.662144 34290678 PMC8287256

[59] Awad A, Yeh HY, Ramadan H, Rothrock MJ. 2023. Genotypic characterization, antimicrobial susceptibility and virulence determinants of Campylobacter jejuni and Campylobacter coli isolated from pastured poultry farms. Front Microbiol 14:1271551. doi:10.3389/fmicb.2023.1271551 38029099 PMC10668334

[60] Cody AJ, McCarthy NM, Wimalarathna HL, Colles FM, Clark L, Bowler I, Maiden MCJ, Dingle KE. 2012. A longitudinal 6-year study of the molecular epidemiology of clinical Campylobacter isolates in Oxfordshire, United kingdom. J Clin Microbiol 50:3193–3201. doi:10.1128/JCM.01086-12 22814466 PMC3457434

[61] Woyda R, Oladeinde A, Endale D, Strickland T, Plumblee Lawrence J, Abdo Z. 2023. Virulence factors and antimicrobial resistance profiles of Campylobacter isolates recovered from consecutively reused broiler litter. Microbiol Spectr 11:e0323623. doi:10.1128/spectrum.03236-23 37882583 PMC10871742

[62] Averós X, Estevez I. 2018. Meta-analysis of the effects of intensive rearing environments on the performance and welfare of broiler chickens. Poult Sci 97:3767–3785. doi:10.3382/ps/pey243 29924356 PMC6162358

[63] Sarıca M, Karakoç K, Erensoy K. 2022. Effects of varying group sizes on performance, body defects, and productivity in broiler chickens. Arch Anim Breed 65:171–181. doi:10.5194/aab-65-171-2022 35572012 PMC9097258

[64] El-Tahawy AAS, Taha AE, Sara AA. 2017. Effect of flock size on the productive and economic efficiency of Ross 308 and Cobb 500 broilers. EuropPoultSci 81. doi:10.1399/eps.2017.175

[65] Ghosh S, Majumder D, Goswami R. 2012. Broiler performance at different stocking density. Ind J Animal Res 46:381–384.

[66] Bailie CL, Ijichi C, O’Connell NE. 2018. Effects of stocking density and string provision on welfare-related measures in commercial broiler chickens in windowed houses. Poult Sci 97:1503–1510. doi:10.3382/ps/pey026 29514265

[67] Buijs S, Keeling L, Rettenbacher S, Van Poucke E, Tuyttens FAM. 2009. Stocking density effects on broiler welfare: identifying sensitive ranges for different indicators. Poult Sci 88:1536–1543. doi:10.3382/ps.2009-00007 19590066

[68] Estevez I, Andersen I-L, Nævdal E. 2007. Group size, density and social dynamics in farm animals. Appl Anim Behav Sci 103:185–204. doi:10.1016/j.applanim.2006.05.025

[69] Greene G, Koolman L, Whyte P, Lynch H, Coffey A, Lucey B, Egan J, O’Connor L, Bolton D. 2021. Maximising productivity and eliminating Campylobacter in broilers by manipulating stocking density and population structure using 'biosecurity cubes'. Pathogens 10:492. doi:10.3390/pathogens10040492 33921776 PMC8073877

[70] Battersby T, Whyte P, Bolton D. 2016. Protecting broilers against Campylobacter infection by preventing direct contact between farm staff and broilers. Food Control 69:346–351. doi:10.1016/j.foodcont.2016.04.053

[71] Simitzis PE, Kalogeraki E, Goliomytis M, Charismiadou MA, Triantaphyllopoulos K, Ayoutanti A, Niforou K, Hager-Theodorides AL, Deligeorgis SG. 2012. Impact of stocking density on broiler growth performance, meat characteristics, behavioural components and indicators of physiological and oxidative stress. Br Poult Sci 53:721–730. doi:10.1080/00071668.2012.745930 23398415

[72] Li W, Wei F, Xu B, Sun Q, Deng W, Ma H, Bai J, Li S. 2019. Effect of stocking density and alpha-lipoic acid on the growth performance, physiological and oxidative stress and immune response of broilers. Asian-Australas J Anim Sci 32:1914–1922. doi:10.5713/ajas.18.0939 31010966 PMC6819680

[73] Tsiouris V, Georgopoulou I, Batzios C, Pappaioannou N, Ducatelle R, Fortomaris P. 2015. High stocking density as a predisposing factor for necrotic enteritis in broiler chicks. Avian Pathol 44:59–66. doi:10.1080/03079457.2014.1000820 25563065

[74] López-López P, Sarmiento-Franco LA, Santos-Ricalde R. 2022. Effect of stocking density on performance, infection by Eimeria spp., intestinal lesions and foot pad injuries in broilers with outdoor access under tropical conditions. Br Poult Sci 63:108–114. doi:10.1080/00071668.2021.1966749 34404284

[75] van der Eijk JAJ, van Harn J, Gunnink H, Melis S, van Riel JW, de Jong IC. 2023. Fast- and slower-growing broilers respond similarly to a reduction in stocking density with regard to gait, hock burn, skin lesions, cleanliness, and performance. Poult Sci 102:102603. doi:10.1016/j.psj.2023.102603 36996512 PMC10070940

[76] Guardia S, Konsak B, Combes S, Levenez F, Cauquil L, Guillot JF, Moreau-Vauzelle C, Lessire M, Juin H, Gabriel I. 2011. Effects of stocking density on the growth performance and digestive microbiota of broiler chickens. Poult Sci 90:1878–1889. doi:10.3382/ps.2010-01311 21844251

[77] Thema KK, Mnisi CM, Mlambo V. 2022. Stocking density-induced changes in growth performance, blood parameters, meat quality traits, and welfare of broiler chickens reared under semi-arid subtropical conditions. PLoS One 17:e0275811. doi:10.1371/journal.pone.0275811 36227929 PMC9560488

[78] Taha-Abdelaziz K, Alkie TN, Hodgins DC, Yitbarek A, Shojadoost B, Sharif S. 2017. Gene expression profiling of chicken cecal tonsils and ileum following oral exposure to soluble and PLGA-encapsulated CpG ODN, and lysate of Campylobacter jejuni. Vet Microbiol 212:67–74. doi:10.1016/j.vetmic.2017.11.010 29173590

[79] Chagneau S, Gaucher M-L, Fravalo P, Thériault WP, Thibodeau A. 2023. Intestinal colonization of Campylobacter jejuni and its hepatic dissemination are associated with local and systemic immune responses in broiler chickens. Microorganisms 11:1677. doi:10.3390/microorganisms11071677 37512849 PMC10385864

[80] Connerton PL, Richards PJ, Lafontaine GM, O’Kane PM, Ghaffar N, Cummings NJ, Smith DL, Fish NM, Connerton IF. 2018. The effect of the timing of exposure to Campylobacter jejuni on the gut microbiome and inflammatory responses of broiler chickens. Microbiome 6:88. doi:10.1186/s40168-018-0477-5 29753324 PMC5948730

[81] Eyerich S, Eyerich K, Cavani A, Schmidt-Weber C. 2010. IL-17 and IL-22: siblings, not twins. Trends Immunol 31:354–361. doi:10.1016/j.it.2010.06.004 20691634

[82] Helmy YA, Closs G, Jung K, Kathayat D, Vlasova A, Rajashekara G. 2022. Effect of probiotic E. coli nissle 1917 supplementation on the growth performance, immune responses, intestinal morphology, and gut microbes of Campylobacter jejuni infected chickens. Infect Immun 90:e0033722. doi:10.1128/iai.00337-22 36135600 PMC9584303

[83] Rzeznitzeck J, Breves G, Rychlik I, Hoerr FJ, von Altrock A, Rath A, Rautenschlein S. 2022. The effect of Campylobacter jejuni and Campylobacter coli colonization on the gut morphology, functional integrity, and microbiota composition of female turkeys. Gut Pathog 14:33. doi:10.1186/s13099-022-00508-x 35922874 PMC9347085

[84] Munoz LR, Bailey MA, Krehling JT, Bourassa DV, Hauck R, Pacheco WJ, Chaves-Cordoba B, Chasteen KS, Talorico AA, Escobar C, Pietruska A, Macklin KS. 2023. Effects of dietary yeast cell wall supplementation on growth performance, intestinal Campylobacter jejuni colonization, innate immune response, villus height, crypt depth, and slaughter characteristics of broiler chickens inoculated with Campylobacter jejuni at d 21. Poult Sci 102:102609. doi:10.1016/j.psj.2023.102609 36963334 PMC10060741

[85] Ducatelle R, Goossens E, De Meyer F, Eeckhaut V, Antonissen G, Haesebrouck F, Van Immerseel F. 2018. Biomarkers for monitoring intestinal health in poultry: present status and future perspectives. Vet Res 49:43. doi:10.1186/s13567-018-0538-6 29739469 PMC5941335

[86] Gilani S, Howarth GS, Kitessa SM, Tran CD, Forder REA, Hughes RJ. 2017. Intestinal permeability induced by lipopolysaccharide and measured by lactulose, rhamnose and mannitol sugars in chickens. Animal 11:1174–1179. doi:10.1017/S1751731116002470 27881199

[87] White RG, Henderson DC, Eslami MB, Neilsen KH. 1975. Localization of a protein antigen in the chicken spleen. Effect of various manipulative procedures on the morphogenesis of the germinal centre. Immunology 28:1–21. 46839 PMC1445742

[88] Igyártó B-Z, Magyar A, Oláh I. 2007. Origin of follicular dendritic cell in the chicken spleen. Cell Tissue Res 327:83–92. doi:10.1007/s00441-006-0250-0 16941124

[89] Cayrou C, Jones M, Williams LK, Chick H, Peeters M, Vermeij P, Frantzen IM, Bijlsmad J, Rushton SP, Humphrey TJ, Sparks NHC, Khattak F, Wilkinson TS, Ketley JM, Bayliss CD. 2024. Contributions of phase variation to host colonisation and extraintestinal spread of multiple Campylobacter jejuni lineages. Appl Environ Microbiol

[90] Chick HM, Rees ME, Lewis ML, Williams LK, Bodger O, Harris LG, Rushton S, Wilkinson TS. 2024. Using the traditional ex vivo whole blood model to discriminate bacteria by their inducible host responses. Biomedicines 12:724. doi:10.3390/biomedicines12040724 38672079 PMC11047930

[91] Lu J, Idris U, Harmon B, Hofacre C, Maurer JJ, Lee MD. 2003. Diversity and succession of the intestinal bacterial community of the maturing broiler chicken. Appl Environ Microbiol 69:6816–6824. doi:10.1128/AEM.69.11.6816-6824.2003 14602645 PMC262306

[92] Indikova I, Humphrey TJ, Hilbert F. 2015. Survival with a helping hand: Campylobacter and microbiota. Front Microbiol 6:1266. doi:10.3389/fmicb.2015.01266 26617600 PMC4637420

