# Supplementary material for: Campylobacter jejuni ST353 and ST464 cause localized gut inflammation, crypt damage, and extraintestinal spread during large- and small-scale infection in broiler chickens
Source: Appl Environ Microbiol. 2025 Feb 18;91(3):e01614-24. doi: 10.1128/aem.01614-24 (PMC11921347; doi:10.1128/aem.01614-24)
Supplement: Supplemental material — Tables S1 to S7; Figures S1 to S16. [file aem.01614-24-s0001.docx]

**Supplementary Table S1 *Campylobacter jejuni* strains used in this study**

| ****Campylobacter jejuni* strains** | **Sequence type** | **Reference / source** |
| --- | --- | --- |
| **NCTC11168** | **ST-21** | **Skirrow 1977, Parkhill 2000, Refs 46 and 47** |
| **M1** | **ST-45** | **Friis 2010, Ref 45** |
| **LE17, LE55, LE89** | **ST-353** | **Naturally infected chickens, reported in**  **Williams LK (submitted), Ref 37 and 38** |
| **LE34, LE104, LE127, LE142** | **ST-464** |  |

*Nine *Campylobacter jejuni* strains were used in this study, including two positive controls (M1 and NCTC11168) and seven strains from naturally infected chickens characterised as ST353 and ST464.

**Supplementary Table S2: Chicken trials and their parameters**

| **Parameter** | **Experiment 1**  **Replicate 1** | **Experiment 1**  **Replicate 2** | **Experiment 2** |
| --- | --- | --- | --- |
| **Site / laboratory** | **SRUC** | **SRUC** | **Boxmeer** |
| **Total number of chickens** | **495** | **495** | **120** |
| **Number of chickens per treatment group** | **45** | **45** | **20** |
| **Number of chickens sampled / time point** | **10** | **10** | **10** |
| **Number of *Campylobacter jejuni* strains** | **10*** | **10*** | **5**** |
| *****Number of treatment groups** | **11** | **11** | **6** |

*Includes NCTC11168 and M1, x3 ST353 (LE17, LE55, LE89), and x4 ST464 (LE34, LE104, LE127 and LE142) and a mixed treatment of equal amounts of LE17 and LE104.

**Includes M1, x2 ST353 (LE17 and LE55), and x2 ST464 (LE104 and LE142)

***Includes an uninfected negative control where chickens received an equal volume of vehicle (distilled water)

**Supplementary Table S3: Treatment *details and organisation of study pens**

*Each treatment was replicated twice in a randomised complete block design as in Supplementary Figure S1. Colour represents treatment type; yellow-uninfected; orange-ST353; red-ST464; green-control strains; purple-mixed treatment of ST353 and ST464

**Supplementary Table S4: Foot pad and hock burn scores**

| **Percentage foot pad (FPS) and hockburn (HS) scores on day 35** | | | | |
| --- | --- | --- | --- | --- |
| **Treatment** | **Foot pad score (FPS)** | | **Hock Scores (HS)** | |
|  | **Absence**  **FPS (%)** | **Presence FPS (%)** | **Absence**  **HS (%)** | **Presence**  **HS (%)** |
| 1 (Invasive strain) | 85 | 15 | 5^a^ | 95^b^ |
| 2 (Invasive strain) | 75 | 25 | 60^ab^ | 40^ab^ |
| 3 (Invasive strain) | 70 | 30 | 10^a^ | 90^b^ |
| 4 (Invasive strain) | 95 | 5 | 60^ab^ | 40^ab^ |
| 5 (Not-Invasive strain) | 80 | 20 | 40^ab^ | 60^ab^ |
| 6 (Mixed strains) | 85 | 15 | 15^a^ | 85^b^ |
| 7 (Not-Invasive strain) | 90 | 10 | 65^ab^ | 35^ab^ |
| 8 (Not-Invasive strain) | 80 | 20 | 25^a^ | 75^b^ |
| 9 (Positive control) | 80 | 20 | 40^ab^ | 60^ab^ |
| 10 (Positive control) | 85 | 15 | 25^a^ | 75^b^ |
| 11(Negative control) | 75 | 25 | 95^b^ | 5^a^ |
| SED | 13.18 | 13.18 | 18.17 | 18.17 |
| P-value | 0.79 | 0.79 | 0.012 | 0.012 |
| Note: FPS and HS recorded on study day 28 were all 0 (meaning absence of lesions).  Scoring criteria for FPS:  0: No lesion and the skin of the foot pad feel soft to the touch and no swelling  1: lesions <5 mm on the pad  2: lesions >5 mm on the pad  Scoring criteria for HS:  0: No discoloration or lesion:  1: .lesions affecting <10% of the hock  2: lesions affecting >10% of the hock.  Calculations:  **Absence (%) FPS or HS** = ((No of 0 scores observed /Total no of birds scored))*100  **Presence (%) FPS or HS** = ((No of 1 score+ 2 score)/Total no of birds scored)*100 | | | | |

**Supplementary Table S5: Primers and probes used in this study**

| **Target** | **Forward Sequence** | **Reverse Sequence** | **Probe Sequence (Fluorophore)** | **Annealing Temperature (T°)** | **Primer efficiency (%)** |
| --- | --- | --- | --- | --- | --- |
| **28S** | **CGAGATTCCCACTGTCCCTA** | **CGCTGTGAAGAGACATGAG** | **CCTCACCGGGTAAGTGAAAA (Fam-Tam)** | **55** | **111.6** |
| **IFN-γ** | **GTGAAGAAGGTGAAAGATATCATGGA** | **GCTTTGCGCTGGATTCTCA** | **TGGCCAAGCTCCCGATGAACGA (Rox-BHQ_2_)** | **55** | **110.8** |
| **IL-17A** | **GAACTGCCTTGCCTAACAGC** | **TCTTCTCATGGAGCACGTTG** | **AATTAGGAATGCAGCCCCTT (Fam-Tam)** | **59** | **96.7** |
| **IL-22** | **TGGGTTGTCTTCTGCTGTTG** | **CGGTTGTTCTCCCTGATGTT** | **CAGACTCATCGGTCAGCAAA (Hex-BHQ_1_)** | **59** | **101.9** |
| **IL-10** | **CATGCTGCTGGGCCTGAA** | **CGTCTCCTTGATCTGCTTGATG** | **CGACGATTCGGCGCTGTCACC (Fam-Tam)** | **59** | **112.8** |
| **CXCLi1** | **TGGCTCTTCTCCTGATCTCAAG** | **GCACTGGCATCGGAGTTCA** | **TCGCTGAACGTGCTTGAGCCATACCTT (Rox-BHQ_2_)** | **57** | **122.1** |
| **CXCLi2** | **GCCCTCCTCCTGGTTTCAG** | **TGGCACCGCAGCTCATT** | **TCTTTACCAGCGTCCTACCTTGCGACA (Hex-BHQ_2_)** | **55** | **112.6** |
| **TGF-β** | **AGGATCTGCAGTGGAAGTGGAT** | **CCCCGGGTTGTGTTGGT** | **ACCCAAAGGTTATATGGCCAACTTCTGCAT (Fam-Tam)** | **59** | **105.9** |

**Supplementary Table S6: *Campylobacter* positivity in liver both replicates (individual birds)**

**Replicate 1**

Brackets represent percentage

**Replicate 2**

Brackets represent percentage

**Supplementary Table S7: *Campylobacter* positivity in spleen both replicates (individual birds)**

**Replicate 1**

Brackets represent percentage

**Replicate 2**

Brackets represent percentage

**Supplementary Figure S1: Organisation of pen layout**. Each treatment (T) was restricted to one pen (P) and was replicated two times in a randomised complete block design. The layout contained 2 blocks (labelled 1, 2). Each block contained two banks (labelled A, B, C and D). Each bank contained treatment pens on the left or right (L, R) side. To maintain maximum stocking density (38kg/m^2^) each pen of 4 m^2^ contained 45 birds each.

**Supplementary Figure S2: *Campylobacter* caecal load in large scale trial experiment 1, replicate 1 (individual strains)**

Ross 308 broilers were challenged with ~10^5^ cfu *C. jejuni* (Table 1 and 2) by direct intubation with the uninfected control group given distilled water. At 7 and 14 dpi, 10 birds in each treatment were randomly selected, humanely killed and a faecal sample from the caecum taken for enumeration of *Campylobacter* following serial dilution and plating. Data is presented as a box and whisker plot where the box defines the median and upper/lower interquartile values, and the whiskers confirm the range. Data values represent log *Campylobacter* counts in caecal samples. Large scale trials plots include an uninfected control (U) and two positive controls, M1 and 11168. *Campylobacter* test strains included; LE17, LE55, LE89 (ST353) and LE104, LE142, LE34 and LE127 (ST464) together with a 50:50 mix of LE17 and LE104. Significant differences between groups were calculated using a 2-way ANOVA and Tukey’s post hoc test. A p<0.05 was considered significant. Symbols correspond to *p<0.05, ** p<0.01, ***p<0.001 and ****p<0.0001. A red* denotes that all infection groups were significantly different to uninfected groups at each time point.

**Supplementary Figure S3: *Campylobacter* caecal load in large scale trial experiment 1, replicate 2 (individual strains)**

Ross 308 broilers were challenged with ~10^5^ cfu *C. jejuni* (Table 1 and 2) by direct intubation with the uninfected control group given distilled water. At 7 and 14 dpi, 10 birds in each treatment were randomly selected, humanely killed and a faecal sample from the caecum taken for enumeration of *Campylobacter* following serial dilution and plating. Data is presented as a box and whisker plot where the box defines the median and upper/lower interquartile values, and the whiskers confirm the range. Data values represent log *Campylobacter* counts in caecal samples. Large scale trials plots include an uninfected control (U) and two positive controls, M1 and 11168. *Campylobacter* test strains included; LE17, LE55, LE89 (ST353) and LE104, LE142, LE34 and LE127 (ST464) together with a 50:50 mix of LE17 and LE104. Significant differences between groups were calculated using a 2-way ANOVA and Tukey’s post hoc test. A p<0.05 was considered significant. Symbols correspond to *p<0.05, ** p<0.01, ***p<0.001 and ****p<0.0001. A red* denotes that all infection groups were significantly different to uninfected groups at each time point.

**Supplementary Figure S4: *Campylobacter* ileal load in large scale trial experiment 1, replicate 1 (individual strains)**

Ross 308 broilers were challenged with ~10^5^ cfu *C. jejuni* (Table 1 and 2) by direct intubation with the uninfected control group given distilled water. At 7 and 14 dpi, 10 birds in each treatment were randomly selected, humanely killed and a faecal sample from the ileum taken for enumeration of *Campylobacter* following serial dilution and plating. Data is presented as a box and whisker plot where the box defines the median and upper/lower interquartile values, and the whiskers confirm the range. Data values represent log *Campylobacter* counts in ileal samples. Large scale trials plots include an uninfected control (U) and two positive controls, M1 and 11168. *Campylobacter* test strains included; LE17, LE55, LE89 (ST353) and LE104, LE142, LE34 and LE127 (ST464) together with a 50:50 mix of LE17 and LE104. Significant differences between groups were calculated using a 2-way ANOVA and Tukey’s post hoc test. A p<0.05 was considered significant. Symbols correspond to *p<0.05, ** p<0.01, ***p<0.001 and ****p<0.0001. A red* denotes that all infection groups were significantly different to uninfected groups at each time point.

**Supplementary Figure S5: *Campylobacter* ileal load in large scale trial experiment 1, replicate 2 (individual strains)**

Ross 308 broilers were challenged with ~10^5^ cfu *C. jejuni* (Table 1 and 2) by direct intubation with the uninfected control group given distilled water. At 7 and 14 dpi, 10 birds in each treatment were randomly selected, humanely killed and a faecal sample from the ileum taken for enumeration of *Campylobacter* following serial dilution and plating. Data is presented as a box and whisker plot where the box defines the median and upper/lower interquartile values, and the whiskers confirm the range. Data values represent log *Campylobacter* counts in ileal samples. Large scale trials plots include an uninfected control (U) and two positive controls, M1 and 11168. *Campylobacter* test strains included; LE17, LE55, LE89 (ST353) and LE104, LE142, LE34 and LE127 (ST464) together with a 50:50 mix of LE17 and LE104. Significant differences between groups were calculated using a 2-way ANOVA and Tukey’s post hoc test. A p<0.05 was considered significant. Symbols correspond to *p<0.05, ** p<0.01, ***p<0.001 and ****p<0.0001. A red* denotes that all infection groups were significantly different to uninfected groups at each time point.

**A)**

**B)**

**Supplementary Figure S6: *Campylobacter* liver load in large scale trial both replicates (individual strains)**

Ross 308 broilers were challenged with ~10^5^ cfu *C. jejuni* (Table 1 and 2) by direct intubation with the uninfected control group given distilled water. At 7 and 14 dpi, 10 birds in each treatment were randomly selected, humanely killed and a liver sample taken and homogenised for enumeration of *Campylobacter* following serial dilution and plating. Data is presented as a box and whisker plot where the box defines the median and upper/lower interquartile values, and the whiskers confirm the range. Data values represent log *Campylobacter* counts in ileal samples. A) Large scale trial replicate 1; B) Large scale trial replicate 2. Large scale trials plots include an uninfected control (U) and two positive controls, M1 and 11168. *Campylobacter* test strains included; LE17, LE55, LE89 (ST353) and LE104, LE142, LE34 and LE127 (ST464) together with a 50:50 mix of LE17 and LE104. Significant differences between groups were calculated using a 2-way ANOVA and Tukey’s post hoc test. A p<0.05 was considered significant. Symbols correspond to *p<0.05, ** p<0.01, ***p<0.001 and ****p<0.0001.

**A)**

**B)**

**Supplementary Figure S7: *Campylobacter* spleen load in large scale trial both replicates (individual strains)**

Ross 308 broilers were challenged with ~10^5^ cfu *C. jejuni* (Table 1 and 2) by direct intubation with the uninfected control group given distilled water. At 7 and 14 dpi, 10 birds in each treatment were randomly selected, humanely killed and a spleen sample taken and homogenised for enumeration of *Campylobacter* following serial dilution and plating. Data is presented as a box and whisker plot where the box defines the median and upper/lower interquartile values, and the whiskers confirm the range. Data values represent log *Campylobacter* counts in ileal samples. A) Large scale trial replicate 1; B) Large scale trial replicate 2. Large scale trials plots include an uninfected control (U) and two positive controls, M1 and 11168. *Campylobacter* test strains included; LE17, LE55, LE89 (ST353) and LE104, LE142, LE34 and LE127 (ST464) together with a 50:50 mix of LE17 and LE104. Significant differences between groups were calculated using a 2-way ANOVA and Tukey’s post hoc test. A p<0.05 was considered significant. Symbols correspond to *p<0.05, ** p<0.01, ***p<0.001 and ****p<0.0001. A red* denotes that all infection groups were significantly different to uninfected groups at each time point.

**Supplementary Figure S8: *Campylobacter* caecal load in small scale trial (individual strains)**

Ross 308 broilers were challenged with ~10^5^ cfu *C. jejuni* (Table 1 and 2) by direct intubation with the uninfected control group given distilled water. At 7 and 14 dpi, 10 birds in each treatment were randomly selected, humanely killed and a faecal sample taken from the caecum for enumeration of *Campylobacter* following serial dilution and plating. Data is presented as a box and whisker plot where the box defines the median and upper/lower interquartile values, and the whiskers confirm the range. Data values represent log *Campylobacter* counts in ileal samples. Small-scale trials plots include an uninfected control (U) and M1 positive control. *Campylobacter* test strains included; LE17 and LE55 (ST353) and LE104 and LE142 (ST464). Significant differences between groups were calculated using 2-way ANOVA and Tukey’s post hoc test. A p<0.05 was considered significant. Symbols correspond to *p<0.05, ** p<0.01, ***p<0.001 and ****p<0.0001. A red* denotes that all infection groups were significantly different to uninfected groups at each time point.

**Supplementary Figure S9: *Campylobacter* ileal load in small scale trial (individual strains)**

Ross 308 broilers were challenged with ~10^5^ cfu *C. jejuni* (Table 1 and 2) by direct intubation with the uninfected control group given distilled water. At 7 and 14 dpi, 10 birds in each treatment were randomly selected, humanely killed and a faecal sample taken from the ileum for enumeration of *Campylobacter* following serial dilution and plating. Data is presented as a box and whisker plot where the box defines the median and upper/lower interquartile values, and the whiskers confirm the range. Data values represent log *Campylobacter* counts in ileal samples. Small-scale trials plots include an uninfected control (U) and M1 positive control. *Campylobacter* test strains included; LE17 and LE55 (ST353) and LE104 and LE142 (ST464). Significant differences between groups were calculated using 2-way ANOVA and Tukey’s post hoc test. A p<0.05 was considered significant. Symbols correspond to *p<0.05, ** p<0.01, ***p<0.001 and ****p<0.0001. A red* denotes that all infection groups were significantly different to uninfected groups at each time point.

**Supplementary Figure 10: *Campylobacter* liver load in small scale trial (individual strains)**

Ross 308 broilers were challenged with ~10^5^ cfu *C. jejuni* (Table 1 and 2) by direct intubation with the uninfected control group given distilled water. At 7 and 14 dpi, 10 birds in each treatment were randomly selected, humanely killed and a sample of liver taken and homogenised for enumeration of *Campylobacter* following serial dilution and plating. Data is presented as a box and whisker plot where the box defines the median and upper/lower interquartile values, and the whiskers confirm the range. Data values represent log *Campylobacter* counts in ileal samples. Table represent a count of *Campylobacter*-positive chickens calculated from ‘direct counts’ with percentages shown in brackets. Small-scale trials plots include an uninfected control (U) and M1 positive control. *Campylobacter* test strains included; LE17 and LE55 (ST353) and LE104 and LE142 (ST464). Significant differences between groups were calculated using a 2-way ANOVA and Tukey’s post hoc test. A p<0.05 was considered significant. Symbols correspond to *p<0.05, ** p<0.01, ***p<0.001 and ****p<0.0001.

**Supplementary Figure S11: *Campylobacter* spleen load in small scale trial (individual strains)**

Ross 308 broilers were challenged with ~10^5^ cfu *C. jejuni* (Table 1 and 2) by direct intubation with the uninfected control group given distilled water. At 7 and 14 dpi, 10 birds in each treatment were randomly selected, humanely killed and a sample of spleen taken and homogenised for enumeration of *Campylobacter* following serial dilution and plating. Data is presented as a box and whisker plot where the box defines the median and upper/lower interquartile values, and the whiskers confirm the range. Data values represent log *Campylobacter* counts in ileal samples. Small-scale trials plots include an uninfected control (U) and M1 positive control. *Campylobacter* test strains included; LE17 and LE55 (ST353) and LE104 and LE142 (ST464). Significant differences between groups were calculated using a 2-way ANOVA and Tukey’s post hoc test. A p<0.05 was considered significant. Symbols correspond to *p<0.05, ** p<0.01, ***p<0.001 and ****p<0.0001.


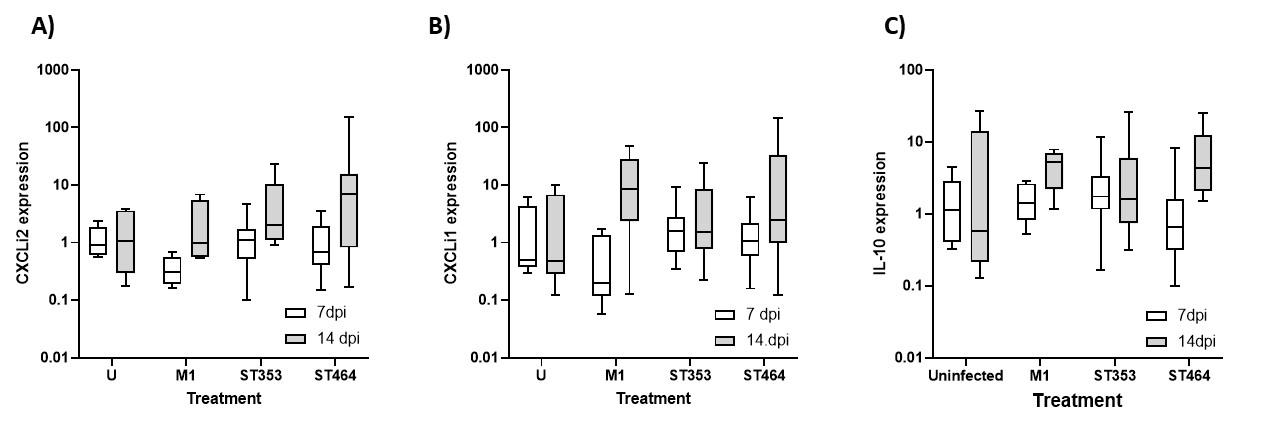


**Supplementary Figure S12 Caecal tonsil levels of CXCLi1, CXCLi2 and IL-10 in large-scale chicken infection trials**

Ross 308 broilers were challenged with ~10^5^ cfu *C. jejuni* (Table 1 and 2) by direct intubation with the uninfected control group given distilled water. At 7 and 14 dpi, 10 birds in each treatment were randomly selected, humanely killed and a sample of caecal tonsil was taken stored in RNAlater prior to RNA isolation and PCR analysis for CXCLi1, CXCLi2 and IL-10 transcripts. Data is presented as a box and whisker plot where the box defines the median and upper/lower interquartile values and the whiskers confirm the range. Data values represent log transformed gene expression using the Pfaffl method. Samples were used from the first large scale trial only and up to 6 chickens were used for each time point. A) CXCLi2 gene expression; B) CXCLi1 gene expression; C) IL-10 gene expression. Plots include an uninfected control (U) and the M1 positive control. Then, data from 2 strains (LE17 and LE55) were combined for ST353 and 2 strains (LE104 and LE142) were combined for ST464. No significant differences were identified between groups when calculated using a 2-way ANOVA and Tukey’s post hoc test. A p<0.05 was considered significant.

**Supplementary Figure S13 Blood levels of serum amyloid A (SAA) in large-scale chicken infection trials**

Ross 308 broilers were challenged with ~10^5^ cfu *C. jejuni* (Table 1 and 2) by direct intubation with the uninfected control group given distilled water. At 7 and 14 dpi, 10 birds in each treatment were randomly selected, and a sample of blood taken into a serum tube before being stored at -20°C. SAA was assayed by specific ELISA. Data is presented as a bar plot where bars define the average and the error bars are the standard deviation. Plot include an uninfected control (U) and the M1 positive control. Then, data from 2 strains (LE17 and LE55) were combined for ST353 and 2 strains (LE104 and LE142) were combined for ST464. Significant differences between groups were calculated using 2-way ANOVA and Tukey’s post hoc test. A p<0.05 was considered significant. Symbols correspond to *p<0.05, ** p<0.01, ***p<0.001 and ****p<0.0001.

**Supplementary Figure 14 Quantification of villi height and width in ileal tissue in large scale chicken trial**

Ross 308 broilers were challenged with ~10^5^ cfu *C. jejuni* (Table 1 and 2) by direct intubation with the uninfected control group given distilled water. At 7 and 14 dpi, 10 birds in each treatment were randomly selected, humanely killed and a tissue sample of ileum taken and fixed in 4% (wt/vol) paraformaldehyde in phosphate-buffered saline (PBS) and routinely embedded in paraffin wax. Sections (3 to 5 µm thick) were prepared and stained with hematoxylin and eosin (H&E) using standard protocols. In brief, each gut section preparation was digitally imaged at high resolution. Images were then analysed using Aperio Image Scope-pathology Slide Viewing Software (Version 12.4.6. Leica Biosystems). For each section, 10 villi were measured, and data presented as A) villi height and B) villi width (µm). Combined data included at least 3 chickens per treatment group and included 3 sections per tissue and 10 measurements for each section. Then, data from 2 strains (LE17 and LE55) were combined for ST353 and 2 strains (LE104 and LE142) were combined for ST464. No significant differences were found between groups when calculated using a 2-way ANOVA and Tukey’s post hoc test. A p<0.05 was considered significant.


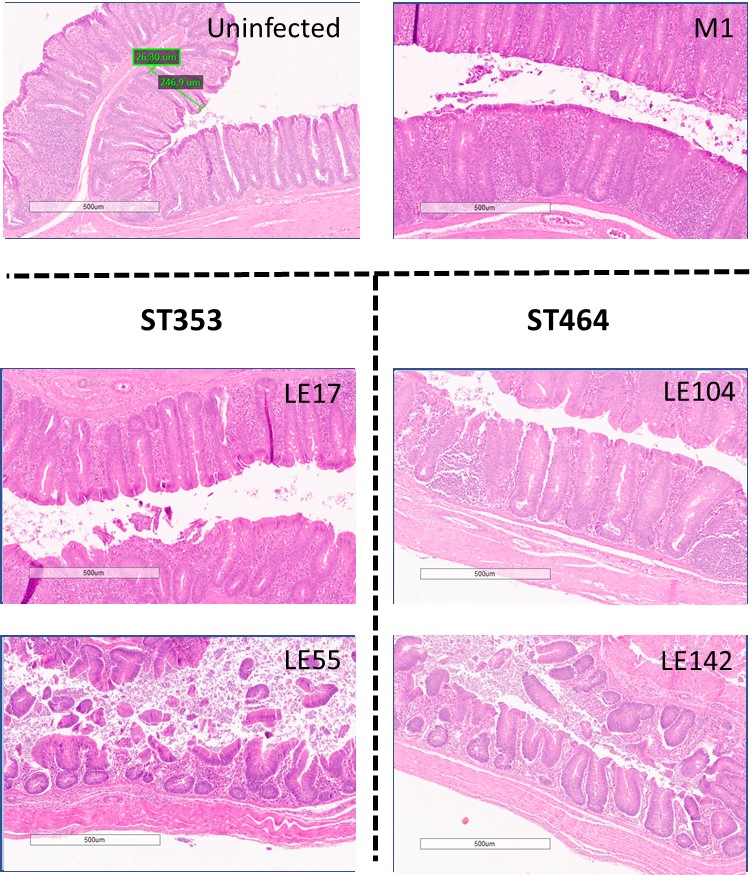


**Supplementary Figure 15A Chicken gut architecture from caecal tissue in large scale chicken trial**

Ross 308 broilers were challenged with ~10^5^ cfu *C. jejuni* (Table 1 and 2) by direct intubation with the uninfected control group given distilled water. At 7 and 14 dpi, 10 birds in each treatment were randomly selected, humanely killed and a tissue sample of ileum taken and fixed in 4% (wt/vol) paraformaldehyde in phosphate-buffered saline (PBS) and routinely embedded in paraffin wax. Sections (3 to 5 µm thick) were prepared and stained with hematoxylin and eosin (H&E) using standard protocols. In brief, each gut section preparation was digitally imaged at high resolution. Images were then analysed using Aperio Image Scope-pathology Slide Viewing Software (Version 12.4.6. Leica Biosystems). Representative images of caecal tissue are shown at 7dpi from uninfected, positive control, M1 and two strains from ST353 and ST464 respectively.


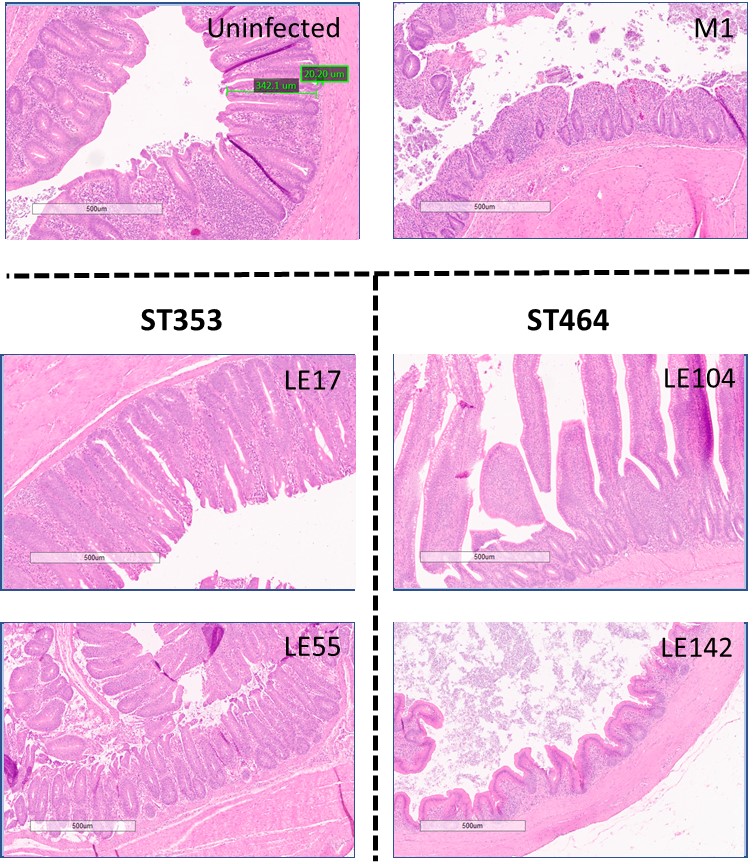


**Supplementary Figure 15B Chicken gut architecture from caecal tissue in large scale chicken trial**

Ross 308 broilers were challenged with ~10^5^ cfu *C. jejuni* (Table 1 and 2) by direct intubation with the uninfected control group given distilled water. At 7 and 14 dpi, 10 birds in each treatment were randomly selected, humanely killed and a tissue sample of ileum taken and fixed in 4% (wt/vol) paraformaldehyde in phosphate-buffered saline (PBS) and routinely embedded in paraffin wax. Sections (3 to 5 µm thick) were prepared and stained with hematoxylin and eosin (H&E) using standard protocols. In brief, each gut section preparation was digitally imaged at high resolution. Images were then analysed using Aperio Image Scope-pathology Slide Viewing Software (Version 12.4.6. Leica Biosystems). Representative images of caecal tissue are shown at 14dpi from uninfected, positive control, M1 and two strains from ST353 and ST464 respectively.


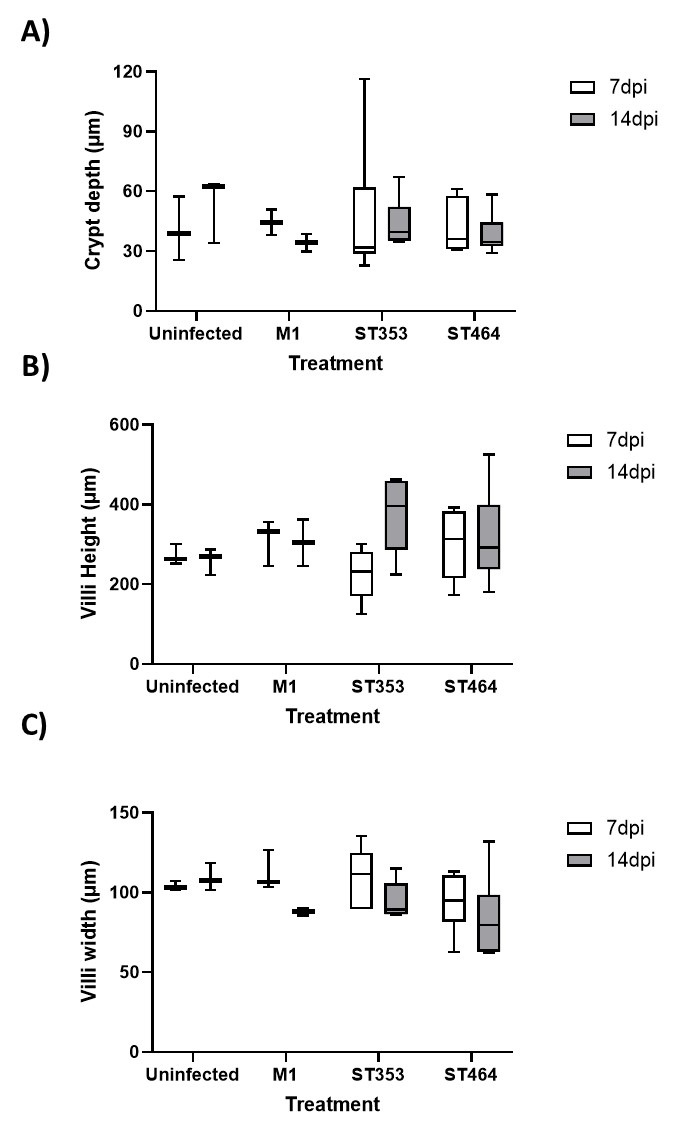


**Supplementary Figure 16 Quantification villi height and width and crypt depth in caecal tissue in large scale chicken trial**

Ross 308 broilers were challenged with ~10^5^ cfu *C. jejuni* (Table 1 and 2) by direct intubation with the uninfected control group given distilled water. At 7 and 14 dpi, 10 birds in each treatment were randomly selected, humanely killed and a tissue sample of caecum taken and fixed in 4% (wt/vol) paraformaldehyde in phosphate-buffered saline (PBS) and routinely embedded in paraffin wax. Sections (3 to 5 µm thick) were prepared and stained with hematoxylin and eosin (H&E) using standard protocols. In brief, each gut section preparation was digitally imaged at high resolution. Images were then analysed using Aperio Image Scope-pathology Slide Viewing Software (Version 12.4.6. Leica Biosystems). For each section, 10 villi were measured, and data presented as A) Crypt depth, B) villi height and C) villi width (µm). Combined data included at least 3 chickens per treatment group and included 3 sections per tissue and 10 measurements for each section. Then, data from 2 strains (LE17 and LE55) were combined for ST353 and 2 strains (LE104 and LE142) were combined for ST464. No significant differences were found between groups when calculated using 2-way ANOVA and Tukey’s post hoc test. A p<0.05 was considered significant.
